# MyD88 signaling causes autoimmune sialadenitis through formation of high endothelial venules and upregulation of LTβ receptor-mediated signaling

**DOI:** 10.1038/s41598-018-32690-x

**Published:** 2018-09-24

**Authors:** Takeshi Into, Shumpei Niida, Ken-ichiro Shibata

**Affiliations:** 1 Department of Oral Microbiology, Division of Oral Infections and Health Sciences, Asahi University School of Dentistry, Mizuho, Japan; 2 Medical Genome Center, National Center for Geriatrics and Gerontology, Obu, Japan; 3 Laboratory of Oral Molecular Microbiology, Department of Oral Pathobiological Science, Hokkaido University Graduate School of Dental Medicine, Sapporo, Japan

## Abstract

Autoimmune sialadenitis (AS), chronic inflammation of the salivary glands (SGs) with focal lymphocyte infiltration, appears in autoimmune diseases such as Sjӧgren’s syndrome. The pathological role of MyD88-dependent innate immune signaling in autoimmune diseases including AS has been studied using mouse models, such as NOD mice. Although AS development in NOD mice was reported to be suppressed by *Myd88* deficiency, its specific role remains unclear. Here, we determined the potent suppressive effects of *Myd88* deficiency on AS development in lupus-prone B6/*lpr* mice, which have lymphoproliferation abnormalities, and also in NOD mice, which have no lymphoproliferation abnormalities. This indicates that MyD88 signaling triggers AS through both lymphoproliferation-dependent and -independent mechanisms. To address the MyD88-dependent lymphoproliferation-independent AS manifestation, SGs from C57BL/6 mice were analyzed. Remarkable upregulation of *Glycam1* and high endothelial venule (HEV)-associated changes were unexpectedly found in *Myd88*^+/+^ mice, compared with *Myd88*^−/−^ mice. MyD88-dependent HEV-associated changes were also observed in NOD mice. Additionally, *Lta*, *Ltb*, and *Ltbr* in SGs of NOD mice were lowered by *Myd88* deficiency. Interestingly, LTβR-induced HEV-associated gene expression in cultured cells was impaired by *Myd88* deficiency. Our findings highlight novel roles for MyD88 in AS development, which imply the existence of MyD88-dependent HEV formation in ectopic lymphoid neogenesis.

## Introduction

Autoimmune sialadenitis (AS) is characterized by chronic inflammation and swelling of the major or minor salivary glands (SGs), along with focal lymphocyte infiltration. In humans, AS is seen in primary Sjögren’s syndrome (SS), and secondary SS associated with other autoimmune diseases such as systemic lupus erythematosus (SLE), scleroderma, and rheumatoid arthritis, and in IgG4-related diseases^1–3^. Under AS conditions, the parenchyma and ducts of the SGs are targeted for destruction by autoantibodies and infiltrating lymphocytes, ultimately causing insufficient saliva secretion and xerostomia^3,4^. The development of AS is thought to have multiple causative factors, including immune factors, genetic background, hormonal abnormalities, and microbial infections^2–4^. Studies using animal models have proved valuable for analyzing the mechanisms of AS progression and regulation, and for testing novel treatments. Various mouse models of spontaneous AS are currently available^5–8^. The lupus-prone strain MRL/*lpr* and its substrains develop AS that is similar to secondary SS in SLE, and the NOD strain and its substrains are regarded as models of primary SS or secondary SS with autoimmune diabetes^5,8–10^.

During AS development, lymphoid organ-like structures form in SG tissues. This includes compartmentalization of infiltrating T and B cells, germinal centers, and a highly organized vasculature with high endothelial venules (HEVs) and lymphatic vessels^11,12^. Such ectopic lymphoid organ-like structures are called tertiary lymphoid organs (TLOs) because their development closely resembles lymphoid neogenesis of secondary lymphoid organs (SLOs), particularly peripheral lymph nodes (LNs), in terms of cellular composition, organization, and vasculature^11,13^. TLOs are thought to function as local sites of antigen presentation by dendritic cells (DCs), and areas of lymphocyte activation for somatic hypermutation and class switching in B cells, suggesting that they can exacerbate autoimmunity^11,14^. However, the regulatory mechanisms that underlie the initiation and progression of TLO formation in AS are not fully understood.

Increasing evidence suggests that the development of autoimmunity involves innate immune detection of nucleic acids^15–17^. In particular, endosomal Toll-like receptors (TLRs) play a key role in recognizing chromatin- or small nuclear ribonucleoprotein-derived antigens, which contain dsDNA or RNA. TLR ligation activates downstream signaling via the adaptor protein MyD88. This in turn activates transcription factors involved in the production of type I IFNs, proinflammatory cytokines, and other proinflammatory mediators^18,19^, which contribute to the development and progression of autoimmunity^19,20^. Previous reports have shown that deletion of MyD88 can prevent lupus manifestations in mice. *Myd88*-deficient MRL/*lpr* mice show no apparent development of autoimmune nephritis^21^. In this model, B cell-intrinsic MyD88-mediated signaling was shown to cause nephritis, whereas in DCs, it is critical for the development of dermatitis^22^. In addition, germinal center formation and anti-nuclear antibody production requires MyD88-mediated signaling in B cells and DCs in lupus-prone *Lyn*-deficient mice^23^. More recently, AS manifestations in NOD mice were found to be prevented by *Myd88* deficiency^24,25^. *Myd88*^−/−^ NOD mice show impaired lymphocyte infiltration in SGs and decreased production of anti-nuclear antibody compared with *Myd88*^+/+^ NOD mice^24,25^. As is thought to occur in lupus, it is possible that MyD88-mediated signaling is important for B cells and DCs in AS development. The detailed mechanism, or the existence of an alternative mechanism, has not been unveiled yet.

In the present study, we sought to investigate the role of MyD88-mediated signaling in AS development using two AS animal models, B6/*lpr* and NOD mice, and comparing *Myd88*^+/+^ mice with *Myd88*^−/−^ mice. We found that *Myd88* deficiency was able to suppress TLO formation, especially HEV formation-associated gene expression in SGs. Furthermore, we found evidence to suggest that activation of lymphotoxin (LT) β receptor (LTβR) signaling, which is important for lymphoid neogenesis in TLOs^26^, is upregulated by MyD88. Our findings highlight a previously unknown role for MyD88 in AS development, and suggest that MyD88-mediated signaling-dependent HEV formation occurs during ectopic lymphoid neogenesis.

## Results

### *Myd88* deficiency suppresses AS development in lupus-prone B6/*lpr* mice

**Figure 1:**
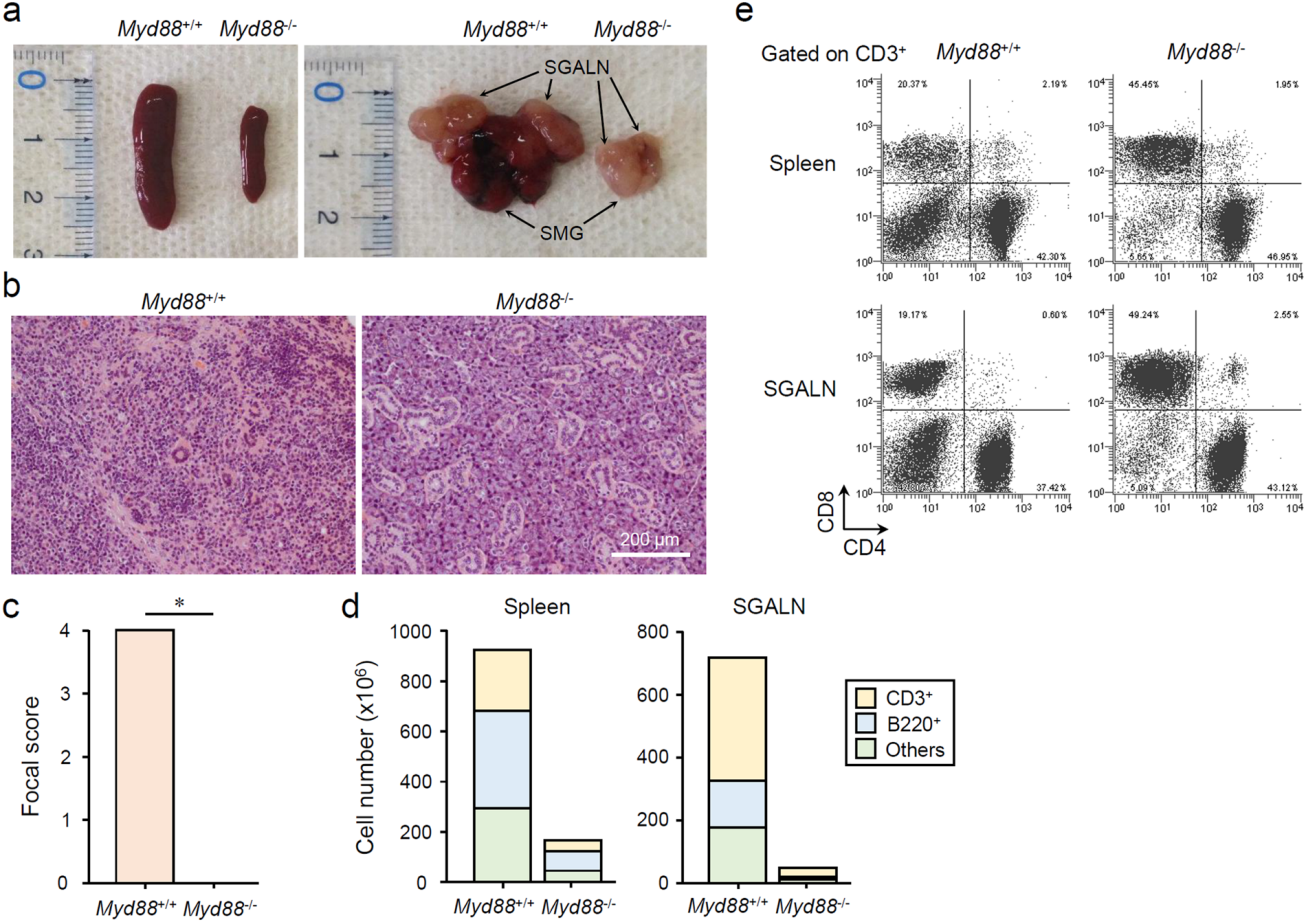
MyD88-dependent AS manifestation and lymphocyte abnormalities in B6/*lpr* mice. (**a**) SLOs and SGs extracted from 24-week-old female *Myd88*^+/+^ and *Myd88*^−/−^ B6/*lpr* mice. Representative spleen (left picture) and SMGs with SGALNs (right picture) for each genotype are shown. (**b**) Representative areas of H&E-stained sections of SGs from *Myd88*^+/+^ (left) and *Myd88*^−/−^ (right) B6/*lpr* mice. Original magnification: ×20. (**c**) Pathological scores of SG sections evaluated from eight mice per genotype. Results are expressed as mean ± SD calculated from mean scores of four sections per animal. *p < 0.01. (**d**) Total number of CD3^+^, B220^+^, and CD3^−^B220^−^ cells collected from spleen (left graph) and SGALN (right graph) from *Myd88*^+/+^ and *Myd88*^−/−^ B6/*lpr* mice was calculated using flow cytometry. Similar results were obtained from all four animals for each strain, therefore a representative result is shown. (**e**) Flow cytometric analysis of CD3^+^ T cells from spleen (upper) and SGALN (lower) of *Myd88*^+/+^ and *Myd88*^−/−^ B6/*lpr* mice. Representative plots for CD4/CD8 expression from three separate experiments are shown.

To determine whether MyD88-mediated signaling affects the development of AS, we compared female *Myd88*^+/+^ B6/*lpr* mice, which spontaneously develop AS along with abnormal lymphoproliferation similar to secondary SS with SLE^5,27^, with *Myd88*^−/−^ B6/*lpr* mice. In 24-week-old female *Myd88*^+/+^ B6/*lpr* mice, severe hyperplasia in the spleen and SG-associated LNs (SGALNs), and swelling of SGs were found (Fig. 1a). In contrast, female *Myd88*^−/−^ B6/*lpr* mice at the same age looked almost normal. The spleen, SGALNs, and SGs of these mice were considerably smaller than those of *Myd88*^+/+^ B6/*lpr* mice (Fig. 1a), and were almost the same as *Myd88*^+/+^ B6 mice (Supplementary Fig. 1). SG histology in *Myd88*^−/−^ B6/*lpr* mice seemed normal, compared with the AS symptoms seen in *Myd88*^+/+^ B6/*lpr* mice, such as severe tissue destruction due to diffuse lymphocyte infiltration (Fig. 1b and c). Large numbers of T and B lymphocytes (CD3^+^ and B220^+^ cells, respectively) could be collected from the spleen and SGALNs of *Myd88*^+/+^ B6/*lpr* mice, whereas the numbers were considerably reduced in *Myd88*^−/−^ B6/*lpr* mice (Fig. 1d). Analysis of T cell subsets revealed an increase in CD3^+^CD4^−^CD8^−^ double-negative T cells in the spleen and SGALNs of *Myd88*^+/+^ B6/*lpr* mice (Fig. 1e). However, in *Myd88*^−/−^ B6/*lpr* mice, the number of double-negative T cells was reduced, and they were shifted towards CD3^+^CD4^−^CD8^+^ T cells (Fig. 1e). Thus, in a lupus-prone AS model that has severe lymphoproliferation, *Myd88* deficiency shows a remarkable suppressive effect on AS development, which is due to a reduction in lymphoproliferation and regulation of lymphocyte differentiation.

### *Myd88* deficiency suppresses AS development in NOD mice

**Figure 2:**
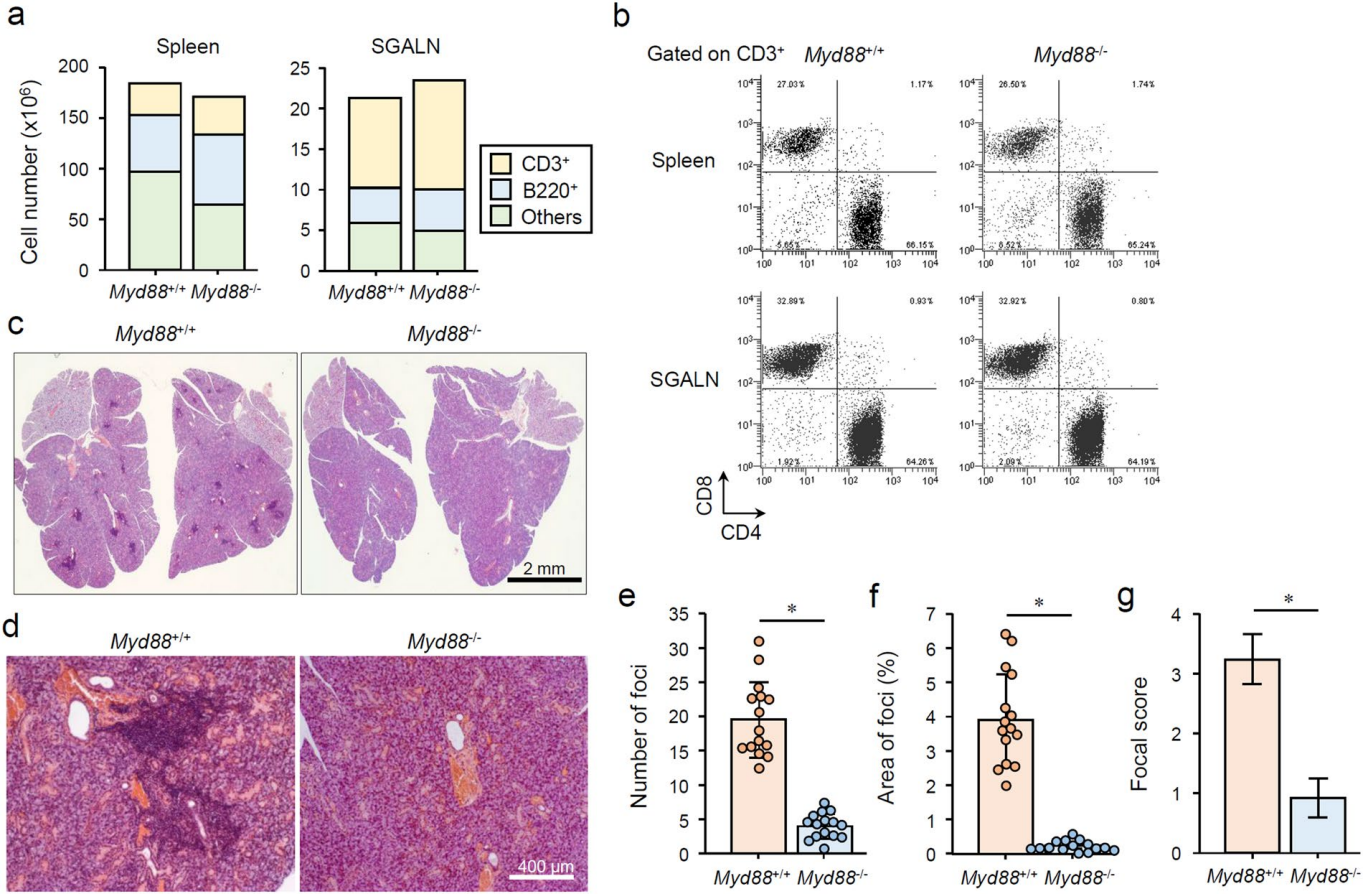
MyD88-dependent AS manifestation in NOD mice. (**a**) Total number of CD3^+^, B220^+^, and CD3^−^B220^−^ cells collected from spleen (left graph) and SGALN (right graph) of 12-week-old female *Myd88*^+/+^ and *Myd88*^−/−^ NOD mice were calculated using flow cytometry. Similar results were obtained from all four animals for each strain, therefore a representative result is shown. (**b**) Flow cytometric analysis of CD3^+^ T cells from spleen (upper) and SGALN (lower) of *Myd88*^+/+^ and *Myd88*^−/−^ NOD mice. Representative plots for CD4/CD8 expression of four separate experiments are shown. (**c**) Representative H&E-stained sections of whole SG from *Myd88*^+/+^ (left) and *Myd88*^−/−^ (right) NOD mice. Original magnification: ×1.5. (**d**) Representative areas of H&E-stained sections of SGs from *Myd88*^+/+^ (left) and *Myd88*^−/−^ (right) NOD mice. Original magnification: ×10. (**e**) Number of foci in SG sections evaluated from fifteen mice per genotype. Results are expressed as mean ± SD (bar graph) of mean values of total foci numbers of four SG sections per animal (circle point). *p < 0.01. (**f**) Percentage of total area of foci in SG sections evaluated from fifteen mice per genotype. Results are expressed as mean ± SD (bar graph) of mean values of percentage of total foci area of four SG sections per animal (circle point). *p < 0.01. (**g**) Focal scores of SG sections evaluated from fifteen mice per genotype. Results are expressed as mean ± SD of mean values of the scores of four SG sections per animal. *p < 0.01.

Next, we investigated the effect of *Myd88*-deficiency on spontaneous AS development in NOD mice. Two groups previously reported that *Myd88* deficiency has a suppressive effect on AS in NOD mice and their substrain^24,25^. As NOD mice do not show severe lymphoproliferation-associated symptoms, including organ hyperplasia, the size of the spleen and SGs was identical in *Myd88*^+/+^ and *Myd88*^−/−^ NOD mice (Supplementary Fig. 2). The number of lymphocytes in the spleen and SGALNs was also approximately the same in *Myd88*^+/+^ and *Myd88*^−/−^ NOD mice (Fig. 2a). Spleen B220^+^ cells were slightly increased in *Myd88*-deficient mice (Fig. 2a), but similar observations have been reported in other mouse strains^28,29^. T cell subsets in the spleen and SGALNs were almost identical in *Myd88*^+/+^ and *Myd88*^−/−^ NOD mice (Fig. 2b).

In NOD mice, the degree of AS development can be evaluated histologically by counting foci number^30^ or by evaluating their area^24^. Foci were present throughout the submandibular glands (SMGs) of 12-week-old female *Myd88*^+/+^ NOD mice, whereas the number of foci, the percentage of total area of foci, and the pathological score were dramatically reduced in *Myd88*-deficient mice (Fig. 2c–g). This is consistent with a previous report using 13-week-old female NOD mice^25^. Thus, *Myd88* deficiency has a suppressive effect on the development of AS, which is not due to lymphoproliferation or lymphocyte differentiation.

### MyD88-dependent TLO-associated changes in SGs from normal female B6 mice

**Figure 3:**
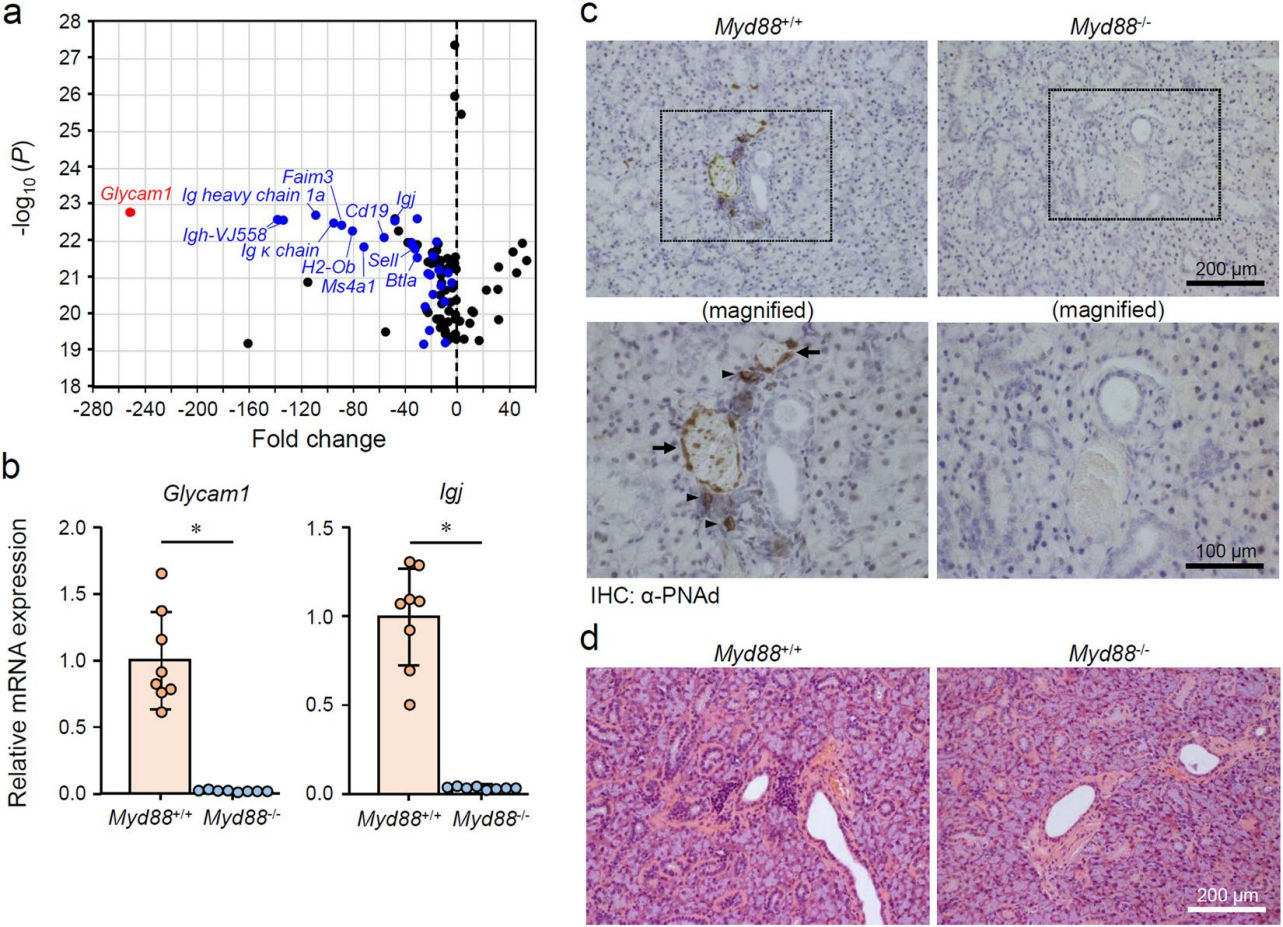
MyD88-dependent TLO formation-associated changes in SGs from normal female B6 mice. (**a**) A volcano plot of the microarray data (top 100 *P* values) of mRNA expression in SMGs from 10-week-old female *Myd88*^−/−^ B6 mice compared with *Myd88*^+/+^ mice (n = 3 per genotype). A red dot indicates *Glycam1*, blue dots indicate B cell- or lymphocyte-associated genes, and black dots represent other genes. The complete list of 100 genes are in Supplementary Table 1. (**b**) qRT-PCR analysis of *Glycam1* and *Igj* expression in whole SMGs from *Myd88*^+/+^ B6 mice and *Myd88*^−/−^ B6 mice (n = 8 per genotype). Expression levels are calculated relative to *Hprt* expression. Results are expressed as mean ± SD (bar graph). *p < 0.01. (**c**) Representative areas of anti-PNAd (MECA-79) antibody-stained sections of SGs from *Myd88*^+/+^ (left) and *Myd88*^−/−^ (right) B6 mice. Arrows and arrowheads indicate HEV-like vessels and probable HEV precursor cells, respectively. Original magnification: ×20 (upper) and ×40 (lower). Additional images are also shown in Supplementary Fig. 4. (**d**) Representative areas of H&E-stained sections of SGs from *Myd88*^+/+^ (left) and *Myd88*^−/−^ (right) B6 mice. Original magnification: ×20. Additional images are also shown in Supplementary Fig. 6.

Our results from two spontaneous AS mouse models indicate that MyD88-mediated signaling contributes to AS development via lymphoproliferation-dependent and -independent mechanisms. The effects of MyD88-mediated signaling on lymphocytes, especially B cells, in autoimmunity have been documented^18,22,23^; therefore, we focused on the lymphoproliferation-independent mechanism. Spontaneous AS mouse models were thought to be unsuitable for the precise assessment of MyD88-dependent and lymphoproliferation-independent initial pathological event in SGs, because the large number of lymphocytes infiltrating in the region may conceal lymphoproliferation-independent events that occur before lymphocytic infiltration. We therefore utilized young female mice on a B6 background because this strain develops AS-like symptoms only in elderly females^31^. To assess the AS-associated initial gene expression profile in SGs, we performed a comprehensive analysis of mRNA expression in the whole SMGs from 10-week-old female *Myd88*^+/+^ and *Myd88*^−/−^ B6 mice. Our results revealed a remarkable reduction in *Glycam1* expression in *Myd88*^−/−^ B6 mice compared with *Myd88*^+/+^ B6 mice (Fig. 3a and Supplementary Table 1). Expression of B cell- or lymphocyte-associated genes, including *Igh-1a*, *Igh-VJ558*, *Igj*, *Sell*, *Faim3*, and *Cd19*, was also lower in SMGs from *Myd88*^−/−^ B6 mice (Fig. 3a and Supplementary Table 1). The changes in *Glycam1* and *Igj* expression were confirmed by individual qRT-PCR analyses (Fig. 3b). Furthermore, the expression of *Glycam1* and *Igj* in SMGs from female mice was considerably higher than in male *Myd88*^+/+^ B6 mice of the same age (data not shown), suggesting that the changes are gender-specific. These observations suggest that the upregulation of *Glycam1* and B cell-associated genes is MyD88-dependent, and this represents the initial gene expression profile of AS development.

GlyCAM-1 (glycosylation-dependent cell adhesion molecule-1; encoded by *Glycam1*) is a ligand for CD62L (also known as L-selectin; encoded by *Sell*)^32,33^. CD62L ligands are constitutively expressed in endothelial cells of HEVs, where they recruit CD62L-expressing naive lymphocytes into lymphoid organs^34^. Other known CD62L ligands, including CD34 (encoded by *Cd34*), podocalyxin (encoded by *Podxl*), endomucin (encoded by *Emcn*), nepmucin (encoded by *Cd300lg*), and MAdCAM-1 (encoded by *Madcam1*), were not significantly affected by *Myd88* deficiency (Supplementary Fig. 3). All CD62L ligands are produced as sulfate-dependent carbohydrate-bound proteins, and are generically referred to as peripheral lymph node addressin (PNAd)^32^. PNAd can be detected using a MECA-79 monoclonal antibody that recognizes an epitope of 6-sulfo Lewis X on the core 1 *O*-glycans^32,35^. We performed immunohistochemistry (IHC) with a MECA-79 antibody and found the formation of few HEV-like structures and infiltration of a small number of PNAd-expressing cells (probably precursors of HEV endothelial cells) in SMGs from female *Myd88*^+/+^ B6 mice, but not in *Myd88*^−/−^ B6 mice (Fig. 3c and Supplementary Fig. 4). Generally, in HEVs, attachment of sulfate-dependent carbohydrates to PNAd is known to be mediated by the *N*-acetylglucosamine-6-*O*-sulfotransferases GlcNAc6ST-1 (encoded by *Chst2*) and GlcNAc6ST-2 (encoded by *Chst4*) and α1,3-fucosyltransferase 4 and 7 (encoded by *Fut4* and *Fut7*)^32,36,37^. We found that the expression of *Chst2*, *Chst4*, and *Fut7* was lower (but *Fut4* was higher) in SMGs from *Myd88*^−/−^ B6 mice compared with that from *Myd88*^+/+^ B6 mice (Supplementary Fig. 5). Furthermore, we observed a low incidence of small, probably non-pathological, regions of lymphocyte infiltration (<50 lymphocytes per focus) in SGs from female *Myd88*^+/+^ B6 mice (Fig. 3d and Supplementary Fig. 6). Such small lymphocyte infiltrates may cause an upregulation in B cell-related genes. Thus, these results indicate that formation of HEVs and small areas of lymphocyte infiltration occurred in SGs from female B6 mice, which are indicative of the initiation of TLO formation^11,38^. Moreover, *MyD88* deficiency exhibits a strong suppressive effect on these processes.

### Verification of TLO formation-associated changes in SGs from NOD mice

**Figure 4:**
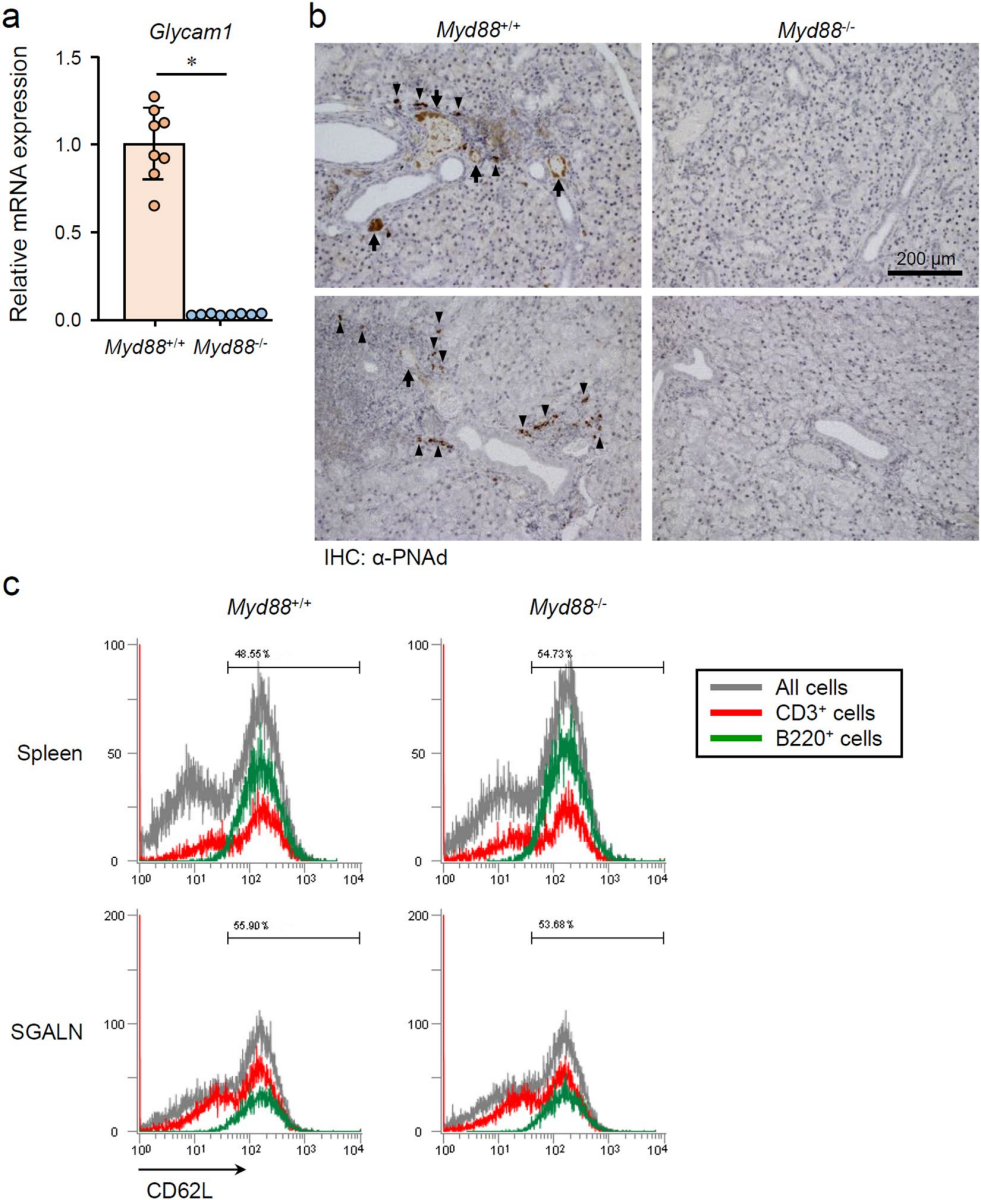
MyD88-dependent HEV formation-associated changes in SGs from NOD mice. (**a**) qRT-PCR analysis of *Glycam1* expression in whole SMG tissues from 12-week-old female *Myd88*^+/+^ and *Myd88*^−/−^ NOD mice (n = 8 per genotype). Expression levels were calculated relative to *Hprt* expression. Results are expressed as mean ± SD (bar graph). *p < 0.01. (**b**) Representative areas of anti-PNAd (MECA-79) antibody-stained sections of SGs from *Myd88*^+/+^ (left) and *Myd88*^−/−^ (right) NOD mice. Arrows and arrowheads indicate HEV-like vessels and probable HEV precursor cells, respectively. Original magnification: ×20. (**c**) Flow cytometric analysis of CD62L in cells prepared from spleen (upper) and SGALNs (lower) from 12-week-old female *Myd88*^+/+^ (left) and *Myd88*^−/−^ (right) NOD mice was performed. Black lines indicate all cells; red lines indicate CD3+ cells, green lines indicate B220+ cells. Results are representative of three independent experiments.

Next, we investigated whether the MyD88-dependent TLO-associated changes occur in NOD mice. qRT-PCR analysis revealed that *Glycam1* expression was remarkably lower in SMGs from 12-week-old *Myd88*^−/−^ NOD mice than from female *Myd88*^+/+^ NOD mice of the same age (Fig. 4a). Additionally, conspicuous formation of HEV-like structures and infiltration of PNAd-expressing cells was found in SMGs of *Myd88*^+/+^ NOD mice, but not in *Myd88*^−/−^ NOD mice (Fig. 4b). On the other hand, formation of HEVs and the presence of PNAd-expressing precursor cells were found in medulla of SGALNs of both *Myd88*^+/+^ and *Myd88*^−/−^ NOD mice (Supplementary Fig. 7), indicating that *MyD88* deficiency does not affect HEV formation in SLOs. GlyCAM-1 serves as a CD62L ligand; therefore, we examined CD62L expression in lymphocytes from *Myd88*^+/+^ and *Myd88*^−/−^ NOD mice. We found no remarkable difference in CD62L expression between lymphocytes collected from the spleen and SGALNs of *Myd88*^+/+^ and *Myd88*^−/−^ NOD mice (Fig. 4c), indicating that *Myd88* deficiency does not affect CD62L expression in lymphocytes.

**Figure 5:**
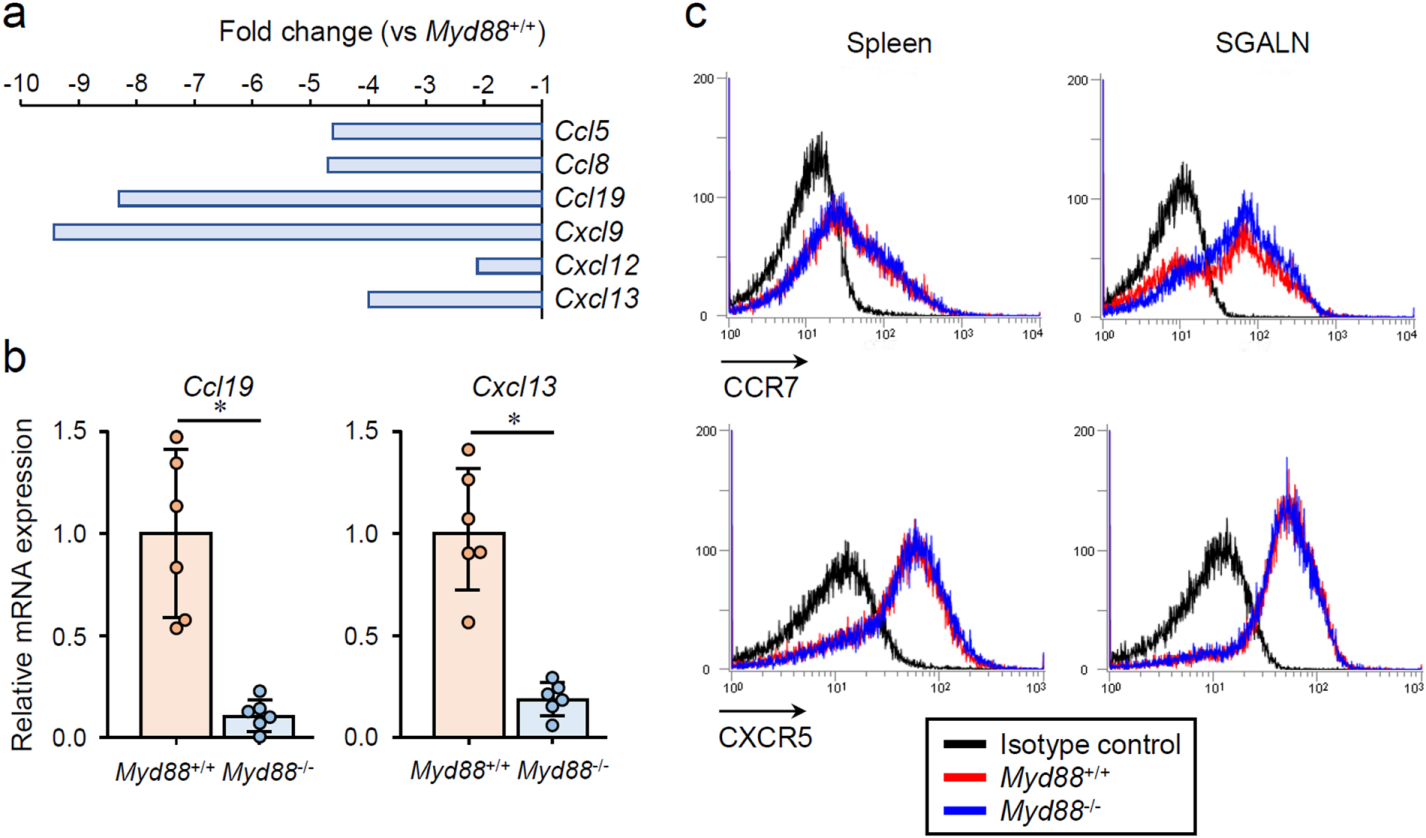
MyD88-dependent expression of lymphoid chemokines in SGs from NOD mice. (**a**) Microarray analysis showing reduced expression of chemokine genes in SMGs from 10-week-old female *Myd88*^−/−^ B6 mice relative to *Myd88*^+/+^ B6 mice (n = 3 per genotype). (**b**) qRT-PCR analysis of *Ccl19* and *Cxcx13* expression in whole SMGs from 12-week-old female *Myd88*^+/+^ and *Myd88*^−/−^ NOD mice (n = 6 per genotype). Expression levels calculated relative to *Hprt* expression. Results are expressed as mean ± SD (bar graph). *p < 0.01. (**c**) Flow cytometric analysis of CCR7 and CXCR5 in cells prepared from spleen (left) and SGALNs (right) from *Myd88*^+/+^ (red lines) and *Myd88*^−/−^ (blue lines) NOD mice. Black lines indicate cells stained with an isotype control antibody from *Myd88*^+/+^ NOD mice. Results are representative of three independent experiments.

A comprehensive analysis of mRNA expression in SMGs from B6 mice revealed a downregulation of several chemokines in *Myd88*^−/−^ mice (Fig. 5a). Among these, CXCL13 and CCL19 are known to be specifically produced by endothelial cells of HEVs, and play an important role in directing naive lymphocytes into lymphoid organs^13,34,39^. qRT-PCR revealed an upregulation of *Cxcl13* and *Ccl19* in SMGs of *Myd88*^+/+^ NOD mice, and that this was impaired by *Myd88* deficiency (Fig. 5b). CCL21 (encoded by *Ccl21a*) is also known to be produced from HEVs to direct lymphocyte entry into lymph nodes^34,39^. However, this chemokine could not be detected (data not shown). Furthermore, we examined the expression of receptors for these chemokines (CCR7 and CXCR5) in lymphocytes collected from spleen or SGALNs and found no obvious difference between *Myd88*^+/+^ and *Myd88*^−/−^ NOD mice (Fig. 5c), indicating that *Myd88* deficiency does not affect the expression of the chemokine receptors CCR7 and CXCR5 in lymphocytes.

### *Myd88* deficiency alters cellular reactivity to LTβR stimulation

**Figure 6:**
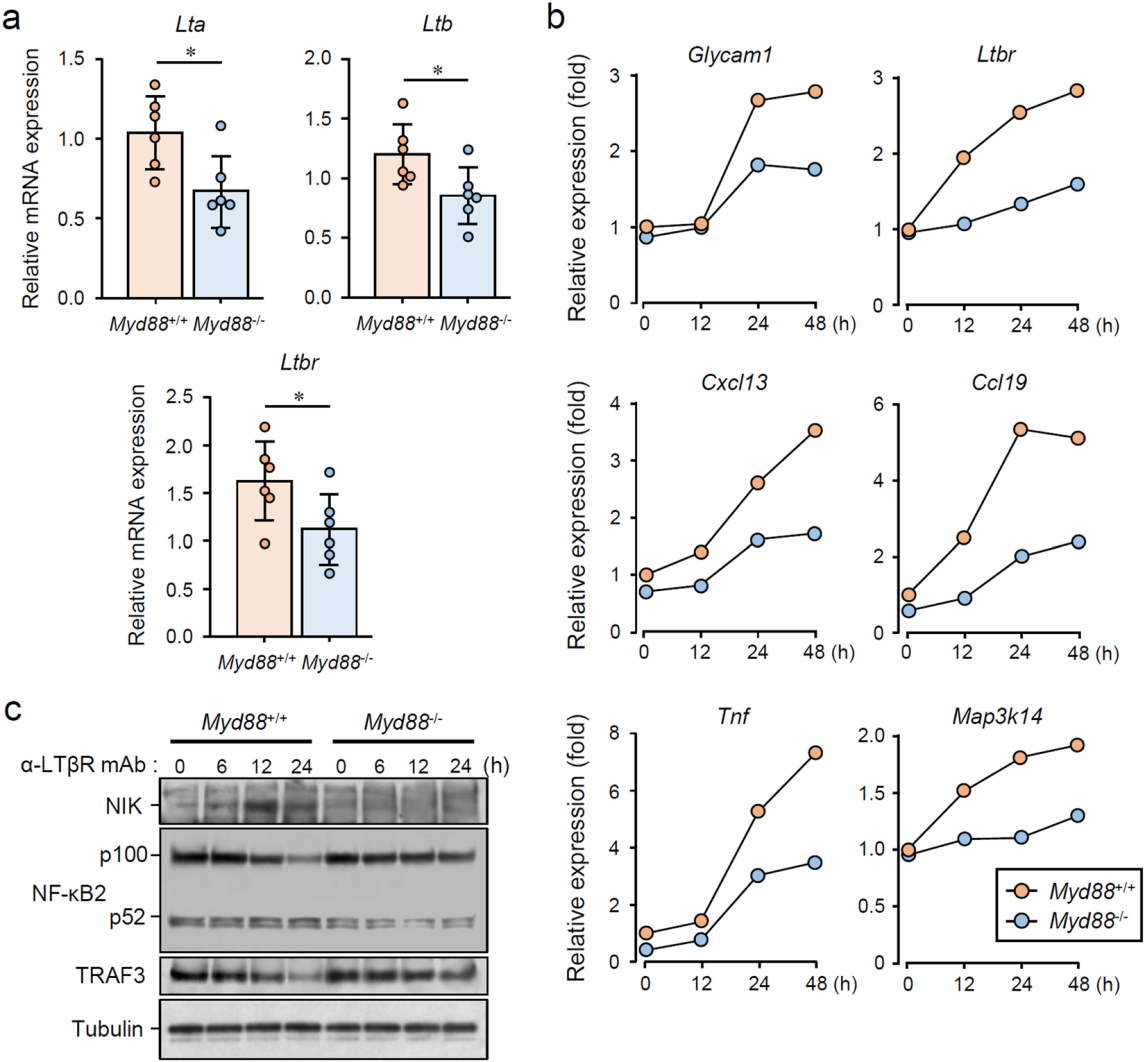
MyD88-dependent LTβR stimulation-induced expression of TLO-associated genes and signal transduction. (**a**) qRT-PCR analysis of *Lta*, *Ltb*, and *Ltbr* expression in whole SMGs from 12-week-old female *Myd88*^+/+^ and *Myd88*^−/−^ NOD mice (n = 6 per genotype). Expression levels calculated relative to *Hprt* expression. Results are expressed as mean ± SD (bar graph). *p < 0.05. (**b**) *Myd88*^+/+^ and *Myd88*^−/−^ MEFs were stimulated with 2.5 μg/mL LTβR agonistic antibody for indicated periods, followed by total RNA extraction. Relative mRNA expression of *Glycam1*, *Cxcl13*, *Ccl19*, *Tnf*, *Map3k14*, and *Ltbr* was determined by qRT-PCR. Expression levels were calculated relative to *Hprt* expression. Representative results of three independent experiments are shown. (**c**) *Myd88*^+/+^ and *Myd88*^−/−^ MEFs were stimulated with 2.5 μg/mL LTβR agonistic antibody for indicated periods, followed by lysis, SDS-PAGE and immunoblotting to assess levels of NIK, NF-κB2 (p100/p52), TRAF3, and Tubulin. Representative results of at least three independent experiments are shown. All the blots were obtained under the same experimental conditions, and the cropped images of the blots are shown. The uncropped images are in Supplementary Fig. 8.

Previous studies reported that administration of an LTβR antagonist to NOD mice results in a remarkable suppression of AS and dacryoadenitis^40,41^. Importantly, this LTβR blockade was found to suppress the expression of *Glycam1* and lymphocyte- associated genes, such as *Igh-1a*, *Sell*, and *Faim3*, in the lesions^40^. Such changes in gene expression are very similar to those caused by *Myd88* deficiency (Fig. 3a), suggesting that there may be a relationship between MyD88-mediated and LTβR signaling. To address this, we first investigated whether expression of the LTβR ligands LTα (encoded by *Lta*) and LTβ (encoded by *Ltb*), and LTβR (encoded by *Ltbr*), can be found in the SGs of NOD mice in a MyD88-dependent manner. qRT-PCR revealed the expression of *Lta*, *Ltb*, and *Ltbr* in SMGs of *Myd88*^+/+^ NOD mice, and that the expression of these genes was significantly lowered by *Myd88* deficiency (Fig. 6a). Next, we stimulated mouse embryonic fibroblasts (MEFs) from *Myd88*^+/+^ and *Myd88*^−/−^ B6 mice with an LTβR-agonistic monoclonal antibody. In *Myd88*^+/+^ MEFs, LTβR stimulation resulted in the induction of *Glycam1*, *Ccl19*, and *Cxcl13* expression, which was attenuated in *Myd88*^−/−^ MEFs (Fig. 6b). Additionally, induction of *Tnf* was also weakened in *Myd88*^−/−^ MEFs compared with that in *Myd88*^+/+^ MEFs (Fig. 6b), suggesting that both canonical and noncanonical NF-κB pathways, which are downstream of LTβR^42–44^, are downregulated by *Myd88* deficiency. We also observed that induction of *Ltbr* (encoding LTβR) and *Map3k14* (encoding NIK, NF-κB-inducing kinase) expression was attenuated by *Myd88* deficiency (Fig. 6b). We further investigated LTβR stimulation-activated signaling events in *Myd88*^+/+^ and *Myd88*^−/−^ MEFs. A slight increase in NIK, activation of NF-κB2 (p100 decrease/p52 increase), and loss of TRAF3 were all observed after LTβR stimulation in *Myd88*^+/+^ MEFs, and this was attenuated by *Myd88* deficiency (Fig. 6c).

To test whether MyD88 is directly recruited by LTβR, we performed several experiments, including co-immunoprecipitation of overexpressed LTβR and confocal analysis of LTβR and MyD88 localization. However, we were unable to obtain any evidence indicating a direct interaction between LTβR with MyD88 (data not shown). Collectively, these results suggest that MyD88-mediated signaling contributes to upregulation of LTβR signaling, but this effect is likely to be indirect.

## Discussion

In the present study, we investigated the role of MyD88-mediated signaling in AS development using two mouse models, and compared *Myd88*^+/+^ mice with *Myd88*^−/−^ mice. In lupus-prone B6/*lpr* mice that have abnormalities in lymphoproliferation and lymphocyte differentiation, AS development is suppressed by *Myd88* deficiency and this is accompanied by a remarkable suppression in lymphocyte abnormalities. Consistent with the previous findings^24,25^, *Myd88* deficiency also suppressed AS development in SS-prone NOD mice, which do not have obvious abnormalities in lymphoproliferation. These results indicate that MyD88-mediated signaling affects AS development via lymphoproliferation-dependent and -independent mechanisms. Moreover, in SGs from female B6 mice, the expression of genes involved in TLO development, especially HEV formation and B cell infiltration, was upregulated, and this was dependent on the presence of MyD88. Additionally, we found that the expression levels of *Lta* (encoding LTα), *Ltb* (encoding LTβ), and *Ltbr* (encoding LTβR) in SGs of NOD mice were reduced by *Myd88* deficiency. Moreover, cellular reactivity to LTβR stimulation, which plays an important role in AS development and TLO formation^11,40,41^, was found to be lowered in *Myd88*-deficient cells. Thus, these results strongly suggest that MyD88-mediated signaling is critically involved in multiple mechanisms of AS development or TLO formation.

MyD88-dependent signaling is known to be involved not only in commensal microbiota-activated immune responses^45^, but also in the regulation of commensal microbiota composition^46^. Of note, Hansen *et al*.^25^ previously reported that AS development in NOD mice is largely dependent on the presence of commensal microbiota, using germ-free NOD mice. Additionally, they found that AS development in germ-free NOD mice can be further suppressed in germ-free *Myd88*-deficient NOD mice, which suggested the presence of MyD88-dependent and commensal microbiota-independent mechanisms in AS development^25^. Although we could not clearly demonstrate how our AS mouse models were affected by commensal microbiota, our results on reduced cellular sensitivity against LTβR stimulation in *Myd88*^−/−^ cells represent one of the commensal microbiota-independent mechanisms. Besides, given that MyD88-dependent signaling seems to be associated with TLO formation, future investigations should determine which processes of TLO formation, including HEV formation, can be regulated by the presence of commensal microbiota.

TLOs are an ectopic accumulation of lymphoid cells that arise in chronic inflammation, and are known to arise from lymphoid neogenesis^11^. Various lines of evidence have shown that TLO formation can be promoted through three critical events: expression of lymphogenous cytokines such as the TNF/LT family; HEV development; and lymphoid chemokine production by stromal cells, such as endothelial cells of HEVs^11,38,47^. In the present study, we observed the formation of *Glycam1* (PNAd)-expressing HEVs and upregulation of the chemokines *Ccl19* and *Cxcl13* in SG tissues. Furthermore, these changes are dependent on the presence of MyD88. Given the fact that AS development can be strongly suppressed by blocking LTβR in NOD mice^41^ and by LTα deficiency in IL-14α-transgenic SS-prone mice^48^, the LTβR ligands (major form LTα1β2 and minor form LTα2β1^49^) are thought to be critical for AS development. We actually observed the lowered expression levels of *Lta*, *Ltb*, and *Ltbr* in SGs from *Myd88*^−/−^ NOD mice compared with *Myd88*^+/+^ NOD mice. A previous study reported that CD11c^+^ DCs are a major source of LTβR stimulation for HEV formation in peripheral LNs, and that depletion of DCs results in a loss of PNAd and impaired naive T cell homing^50^. Importantly, LTα and LTβ produced by DCs can stimulate *Glycam1*, *Chst4*, and *Fut7* expression in HEVs^50^. Given that TLOs and SLOs form through similar mechanisms^11,38,47^, it is possible that DCs in SGs produce excessive amounts of LTβR ligands in a MyD88-mediated signaling-dependent manner, which leads to TLO formation in AS. Moreover, cellular reactivity to LTβR stimulation can be enhanced in the presence of MyD88. The detailed mechanism underlying this phenomenon and the role of MyD88-mediated signaling will be the subject of future studies.

The LTβR signaling pathway is broadly involved in the lymphoid neogenesis in both SLOs and TLOs^26,51–53^. LTβR is expressed by various hematopoietic and non-hematopoietic cells, including cells of epithelial and myeloid lineages, but not T and B cells^43^. Signaling through LTβR is activated by ligation of LTα1β2, LTα2β1, or LIGHT, which initiates receptor clustering^43,49^. LTβR subsequently recruits TRAF2, TRAF3 or TRAF5, and activates two gene transcription programs via transcription factor NF-κB: the classical pathway, characterized by nuclear translocation of p50-RelA NF-κB; and the alternative, non-canonical pathway, characterized by NIK (NF-κB-inducing kinase)-dependent activation of IκB kinase (IKK)-α and nuclear translocation of p52-RelB NF-κB^43,44^. Appropriate activation of the non-canonical NF-κB pathway requires induction of A20, which itself occurs through the canonical NF-κB pathway^54^. In the present study, the downstream signaling events of LTβR were reduced in the absence of MyD88, where both the canonical and non-canonical NF-κB pathways were affected. The key event in the non-canonical NF-κB pathway is signal activation-dependent protein stabilization of NIK, which would normally be degraded by a ubiquitin ligase complex comprised of TRAF2, TRAF3, and cIAP1/2^42,44^. NIK stabilization in turn results in degradation of TRAF3. However, this event is transient, and continual activation requires appropriate levels of NIK to sustain the activity of the non-canonical NF-κB pathway^42–44^. We found that LTβR stimulation-induced expression of *Map3k14* (encoding NIK) was impaired by *Myd88* deficiency. It is known that LTβR-induced activation of the non-canonical NF-κB pathway plays a dominant role in HEV formation, as well as in the expression of several HEV-specific genes, including *Glycam1*, and *Chst4*^13,50,55^. This indicates that the changes in the gene expression profiles of SGs from normal B6 mice and NOD mice are mediated by the non-canonical NF-κB pathway. Although a direct interaction of LTβR with MyD88 was not identified in this study, it is possible that an unknown association of LTβR signaling with MyD88-mediated signaling can occur. Indeed, MyD88 was clearly involved in the LTβR-induced earlier expression of several genes, such as *Ltbr* and *Ccl19* (Fig. 6a). Moreover, MyD88 signaling may be activated by a later LTβR-induced production of proinflammatory cytokines, such as IL-1β and IL-18, or TLR-stimulating DAMPs^19,56^, which would ultimately induce MyD88-dependent indirect upregulatory effects on LTβR signaling.

Our results indicate that *Myd88* deficiency strongly suppresses TLO formation, but does not affect the formation of SLOs, including spleen and SGALNs. It is known that the architecture of TLOs closely resembles that of SLOs, particularly peripheral LNs, in terms of cellular composition, organization, chemokine expression, and vasculature^11,38^. TLOs display organized lymphocyte subtype compartmentalization, which is driven by lymphoid chemokines such as CCL19 and CXCL13, the formation of germinal centers, and a highly organized vascular system, including HEVs, lymphatic vessels and conduits^38,47^. Why, then, does MyD88 signaling not affect SLO formation? Although the details are not clear at present, it is possible that different subtypes of DCs are involved in SLO versus TLO development^47,50^. It is also possible that MyD88-dependent upregulation of LTβR signaling may not occur during SLO development. Our results indicate that MyD88-mediated signaling upregulates LTβR signaling in an indirect, rather than direct, manner. Indeed, *Myd88* deficiency does not completely obliterate AS development or cellular reactivity towards LTβR stimulation. Further investigation will be necessary to determine exactly how MyD88 signaling regulates TLO formation.

We found that the expression of HEV formation-related genes, including *Glycam1*, is elevated in SGs from AS model mice. Since this phenomenon was originally observed in SGs from normal B6 female mice, these changes could be regarded as gender-specific and AS-related events. These genes could be potentially used as clinical markers of disease progression and severity, but further studies will be needed to establish their exact role and specificity. Additionally, as TLOs are known to be highly plastic^11,57^, those that form in SGs could be ablated by appropriate medical interference that can remove the initial signs of AS. Although careful consideration is required to ascertain whether targeting of MyD88 *per se* could be a better way to treat TLOs that form in the SGs, the results of the current study, together with those from previous reports, suggest that the molecules associated with MyD88-mediated signaling could be strong candidates for the treatment. A better understanding of the detailed mechanisms is needed to allow precise manipulation of TLO formation, with the aim of conquering autoimmune-induced disorders and generating new therapeutics for AS-related pathologies.

## Methods

### Mice

C57BL/6JJmsSlc (B6) and B6.MRL-*Fas*^lpr/lpr^/Slc (B6/*lpr*) mice were obtained from Japan SLC (Hamamatsu, Japan). NOD/ShiLtJ (NOD) mice were obtained from Charles River Laboratories, Japan (Yokohama, Japan). B6-*Myd88*^−/−^ mice were described previously^29^. A congenic strain, NOD.B6-*Myd88*^−/−^, was generated by crossing NOD with B6-*Myd88*^−/−^ mice. Heterozygotes were backcrossed to NOD for 11 generations, followed by appropriate sister-brother matings to generate NOD.B6-*Myd88*^−/−^ mice. B6/*lpr*-*Myd88*^−/−^ mice were generated by crossing B6/*lpr* with B6-*Myd88*^−/−^ mice. Heterozygotes (*Myd88*^+/−^*Fas*^+/lpr^) were backcrossed to B6/*lpr*, followed by appropriate sister-brother matings to generate B6/*lpr*-*Myd88*^−/−^*Fas*^lpr/lpr^ mice. Mice were genotyped by polymerase chain reaction (PCR) analysis using genomic DNA from tail biopsies and the following oligonucleotide primers: *Myd88* forward (5′-TGGCATGCCTCCATCATAGTTAACC-3′), *Myd88* reverse (5′-GTCAGAAACAACCACCACCATGC-3′), neomycin cassette reverse (5′-ATCGCCTTCTATCGCCTTCTTGACG-3′); *Fas* forward (5′-GTAAATAATTGTGCTTCGTCAG-3′), *Fas* reverse (5′-TAGAAAGGTGCACGGGTGTG-3′) and *Fas*^lpr^ reverse (5′-CAAATCTAGGCATTAACAGTG-3′).

Mice (2 to 4 per cage) were maintained in the animal facility at the Asahi University School of Dentistry. Mice were fed water and a radiation-sterilized diet *ad libitum* with HEPA-filtered air in a conventional animal room (23 ± 2 °C, 50% humidity, 12 h light/dark cycle). All animal studies were approved by the Committee on the Ethics of Animal Experiments of the Asahi University (Permit Numbers: 15-001, 16-008 and 17-015) and were carried out in accordance with approved guidelines. All efforts were made to minimize suffering of animals.

### Histology, foci counting, and focal scoring

Twenty-four-week-old female B6/*lpr* mice, 12-week-old NOD mice and 10-week-old B6 mice were used. Both sides of the major SG and SGALN were comprehensively removed, fixed in 10% formalin and paraffin-embedded. For each animal, at least four sections (5 μm thick) were cut at 100-μm intervals, and stained with H&E. Images of tissue sections were obtained using an SZ stereomicroscope with a DP21 digital camera (Olympus) and a BX41 microscope (Olympus), and processed using Paint.NET (dotPDN LLC and Rick Brewster). Images of four sequential sections per animal were scored by a trained practitioner in a double-blind manner. The score was assigned by counting the total number of foci with infiltrates >50 mononuclear cells and calculating the mean number of foci per animal. The same sequential sections were scored for the degree of lymphocyte infiltration and tissue damage by a trained practitioner in a double-blind manner, based on previously assigned focal scoring criteria^58^. Briefly, a focal score of 1 indicates 1–5 foci (>50 mononuclear cells per focus) per section; 2 indicates more than 5 mononuclear cell foci without significant parenchymal destruction; 3 indicates multiple confluent foci with moderate degeneration of parenchymal tissue; 4 indicates extensive mononuclear cell infiltration with extensive parenchymal destruction. A focal score of 0 means no foci were detected. Lymphocytic infiltration in SG tissues was quantified by the division of the area of infiltration by the total tissue area examined, as described elsewhere^24^. Results were expressed as mean ± standard deviation (SD) calculated from the mean values of four scores per animal.

### Immunohistochemistry (IHC)

Twelve-week-old female NOD mice and 10-week-old B6 mice were used. SG and SGALN sections were prepared as described above, and sections were deparaffinized and immersed in 10 mM citrate buffer (pH 6.0) then autoclaved at 121 °C for 10 min. This was followed by treatment with 3% H_2_O_2_ for 15 min at room temperature and three washes with 0.05 M phosphate buffer (pH 7.6). Sections were blocked with Blocking I reagent (Nacalai tesque) for 10 min followed by incubation overnight at 4 °C with a rat anti-mouse PNAd carbohydrate epitope monoclonal antibody (553863; BD Pharmingen). After three washes with phosphate buffer, sections were incubated with an HRP-conjugated goat anti-rat IgM μ chain preabsorbed antibody (ab98373; Abcam) for 30 min at room temperature. After washing, sections were developed with 3,3′-diaminobenzidine, washed three times, then counterstained with Mayer’s hematoxylin for 1 min. Images were obtained using a BX41 microscope and processed using Paint.NET.

### Flow cytometry

SGALNs or spleens were homogenized using gentleMACS C tubes (Miltenyi Biotech) and a gentleMACS Dissociator (Miltenyi Biotech). Cells were filtered through a 70-μm nylon cell strainer (BD Falcon, Franklin Lakes, NJ, USA), washed with PBS containing 2 mM EDTA and 0.5% BSA (PEB), and resuspended in PEB at 1 × 10^7^ cells/mL. Then, 1 × 10^6^ cells were treated with mouse FcR Blocking Reagent (Miltenyi Biotech) for 10 min at 4 °C followed by incubation with fluorescence-labeled antibodies for 20 min at 4 °C. Cells were washed with PEB, resuspended in 1% paraformaldehyde in PBS, and stored at 4 °C. Samples were analyzed by flow cytometry using an EC800 Cell Analyzer (SONY, Tokyo, Japan) and accompanying software.

Alexa Fluor 488 anti-mouse CD3 (clone 17A2), PE anti-mouse CD3 (clone 17A2), Alexa Fluor 488 anti-mouse CD4 (clone GK1.5), PE/Cy7 anti-mouse CD8a (clone 53-6.7), PE/Cy7 anti-mouse CD62L (clone MEL-14), and PE anti-mouse CCR7 (CD197; clone 4B12) were obtained from BioLegend. PE anti-mouse CXCR5 (CD185; clone 12-7185) was obtained from eBioscience. Fluorescence-labeled isotype-matched control antibodies were obtained from BioLegend.

### Microarray analysis of SMGs

Comprehensive RNA expression in SMGs from 10-week old female B6 mice was analyzed using Agilent Whole Mouse Genome Oligo Microarrays as previously described^29^. The microarray dataset is available from the Gene Expression Omnibus (Accession Number, GSE61339).

### Cell culture

MEFs were prepared from 13.5-day embryos from *Myd88*^+/+^ and *Myd88*^−/−^ B6 mice and cultured as previously described^59^. MEFs (5 × 10^5^ cells) were cultured in 6-well plates and stimulated with 2.5 μg/mL rat anti-mouse LTβR monoclonal antibody 5G11b (low endotoxin product, MCA2244EL; Bio-Rad).

### Quantitative reverse transcription-PCR (qRT-PCR)

SYBR Green-based qRT-PCR using total RNA obtained from SG tissues or cultured MEFs was performed as previously described^29,59^. Primers for mouse *Glycam1*, *Igj*, *Ccl19*, *Cxcl13*, *Tnf*, *Map3k14*, *Ltbr*, and *Hprt* were obtained from QIAGEN (Hilden, Germany). Gene expression was determined using the ΔΔ*C*_*t*_ method. Results were shown as relative expression, and normalized to levels of the housekeeping gene *Hprt*.

### Immunoblotting

Immunoblotting of lysates from cultured MEFs was performed as previously described^59^. Immunoreactive bands were detected using the following primary antibodies and a horseradish peroxidase-conjugated secondary antibody: anti-NIK rabbit polyclonal antibody (4994), anti-NF-κB2 p100/p52 rabbit polyclonal antibody (4882), and anti-α/β-tubulin (2148) rabbit monoclonal antibody, all from Cell Signaling Technology; and anti-TRAF3 rabbit polyclonal antibody (HPA002933) from Sigma-Aldrich. Clarity Western ECL Substrate (Bio-Rad) was used to visualize the blots using an ECL minicamera (Amersham Biosciences) with instant black and white film FP-3000B (Fuji Films). Images were obtained using a GT-S650 scanner (Epson).

### Statistical analysis

Data are expressed as mean ± SD. *P* values were calculated using an unpaired Student’s *t*-test, and those less than 0.05 or 0.01 were considered significant.

## Electronic supplementary material

Supplementary Information
