# Supplementary material for: MyD88 signaling causes autoimmune sialadenitis through formation of high endothelial venules and upregulation of LTβ receptor-mediated signaling
Source: Sci Rep. 2018 Sep 24;8:14272. doi: 10.1038/s41598-018-32690-x (PMC6155371; doi:10.1038/s41598-018-32690-x)
Supplement: Supplementary file 1 — Supplementary Information [file 41598_2018_32690_MOESM1_ESM.pdf]

Supplementary Information

MyD88 signaling causes autoimmune sialadenitis through formation of high endothelial venules and upregulation of LTβ receptor-mediated signaling

Takeshi Into[1]*, Shumpei Niida[2] & Ken-ichiro Shibata[3]

[1]Department of Oral Microbiology, Division of Oral Infections and Health Sciences, Asahi University School of Dentistry, Mizuho, Japan.

[2]Medical Genome Center, National Center for Geriatrics and Gerontology, Obu, Japan.

[3]Laboratory of Oral Molecular Microbiology, Department of Oral Pathobiological Science, Hokkaido University Graduate School of Dental Medicine, Hokkaido, Japan.

*Correspondence should be addressed to T.I. (into@dent.asahi-u.ac.jp)

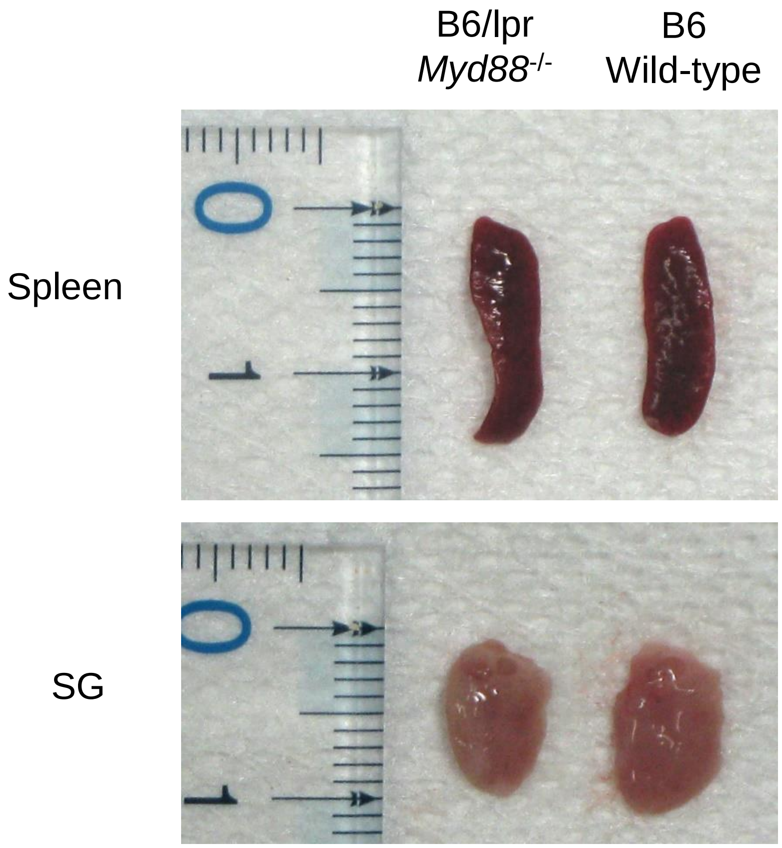

Supplementary Figure 1. Spleens and SGs extracted from *Myd88*-deficient B6/lpr and wild-type C57BL/6 mice.

Representative spleen (upper) and SGs (with SGALNs; lower) collected from 24-week-old female *Myd88*-deficient B6/lpr (left) and wild-type C57BL/6 (right) are shown.

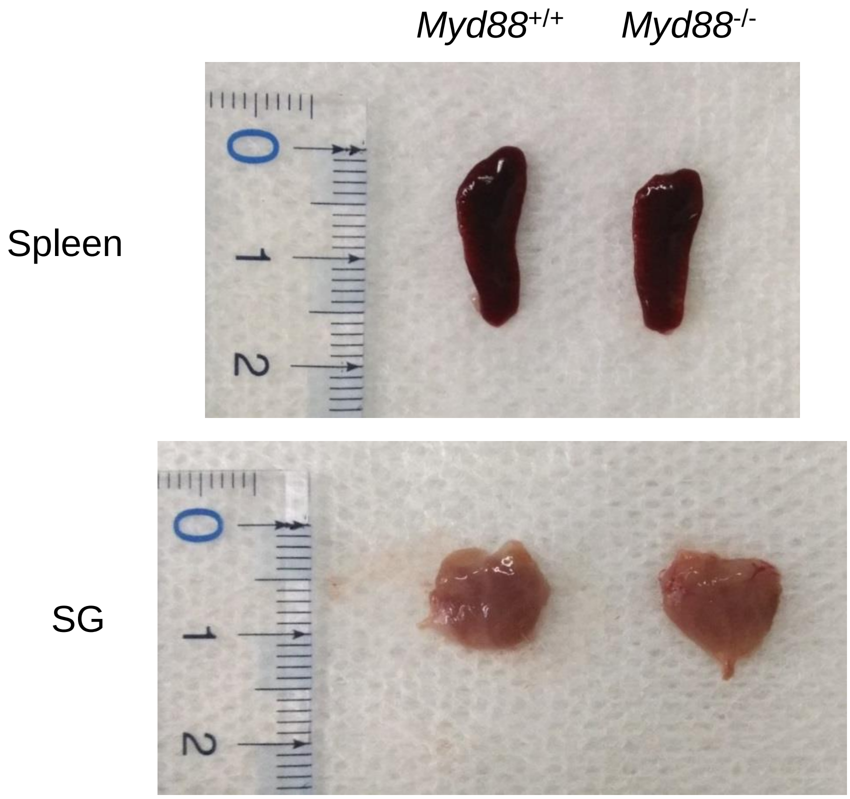

Supplementary Figure 2. Spleens and SGs extracted from wild-type and *Myd88*-deficient NOD mice. Representative spleen (upper) and SGs (with SGALNs; lower) collected from 12-week-old female wild-type NOD (left) and *Myd88*-deficient NOD (right) are shown.

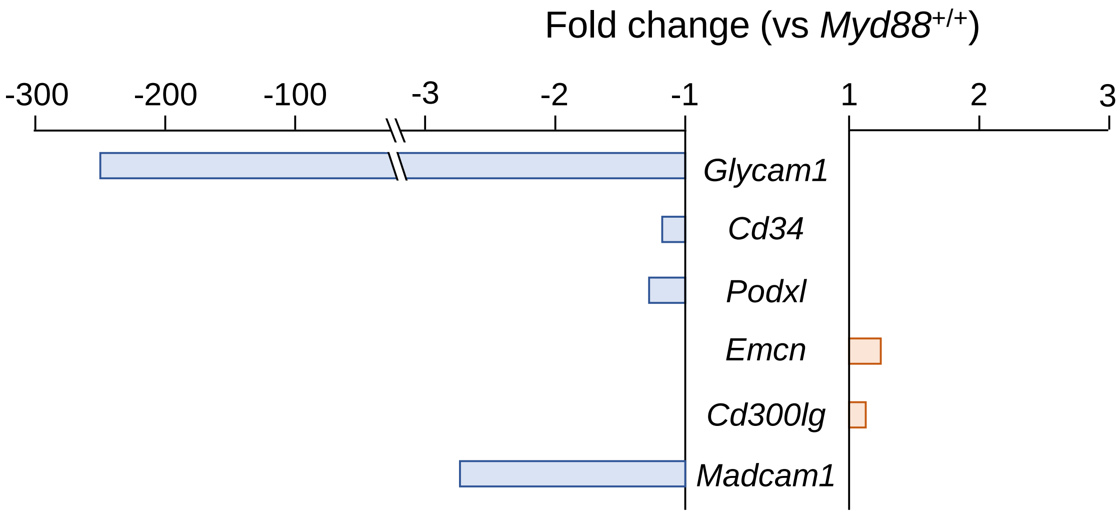

Supplementary Figure 3. Microarray analysis of mRNA expression of HEV-associated CD62L ligands in SMGs from female *Myd88*-deficient B6 mice.

Results show mRNA expression levels of HEV-associated CD62L ligands in SMGs from 10-week-old female *Myd88*-deficient B6 mice relative to those from wild-type mice (n = 3 per genotype).

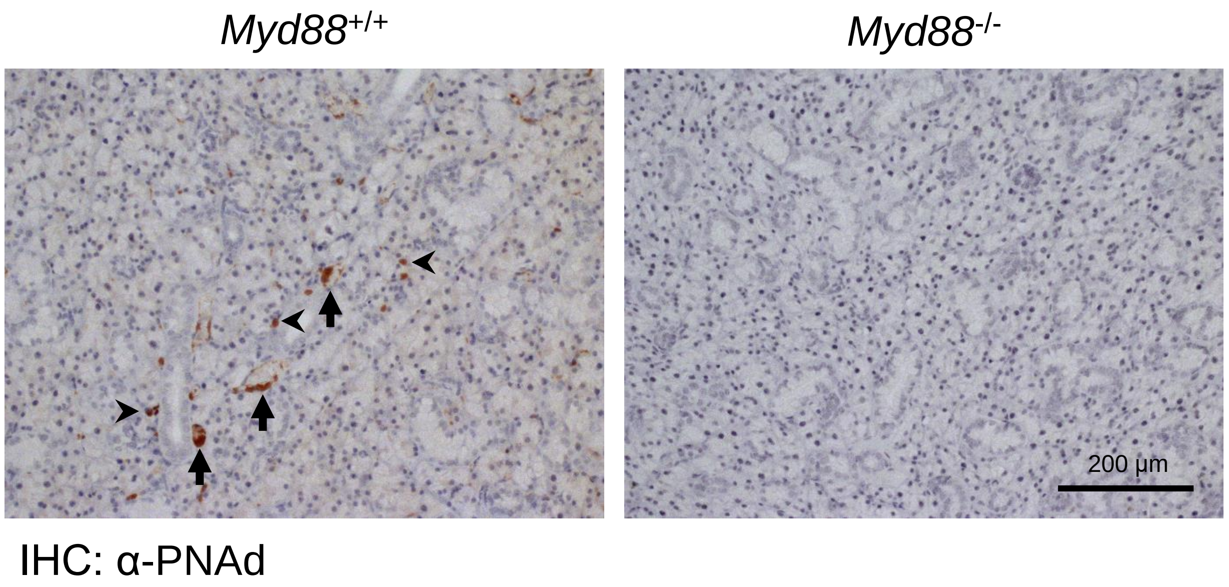

Supplementary Figure 4. Immunohistochemical staining of PNAd in SGs from wild-type and *Myd88*-deficient B6 mice.

Representative areas of anti-PNAd (MECA-79) antibody-stained sections of SGs from 10-week-old female wild-type (left) and *Myd88*-deficient (right) B6 mice. Arrows and arrowheads indicate HEV-like vessels and probable HEV precursor cells, respectively. Original magnification: ×20. Additional images are shown in Fig. 3c.

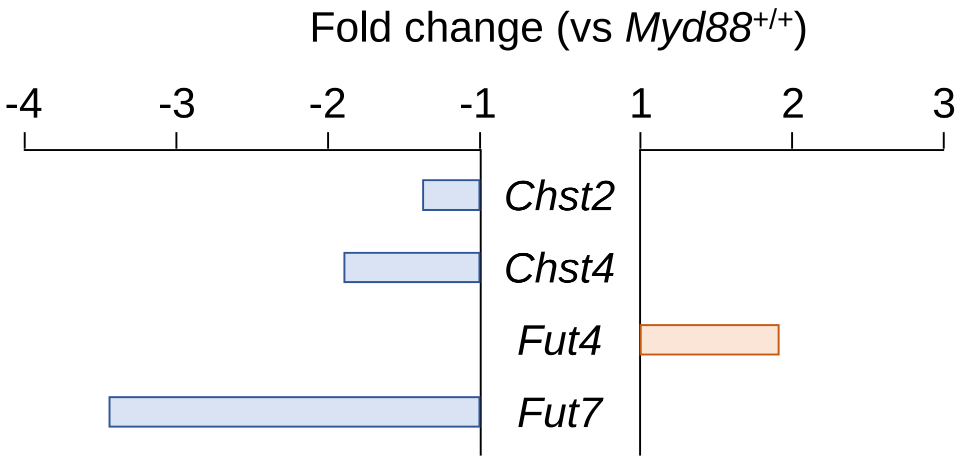

Supplementary Figure 5. Microarray analysis of mRNA expression of HEV-associated enzymes in SMGs from female *Myd88*-deficient B6 mice.

Results show mRNA expression levels HEV-associated enzymes in SMGs from 10-week-old female *Myd88*-deficient B6 mice relative to wild-type (n = 3 per genotype).

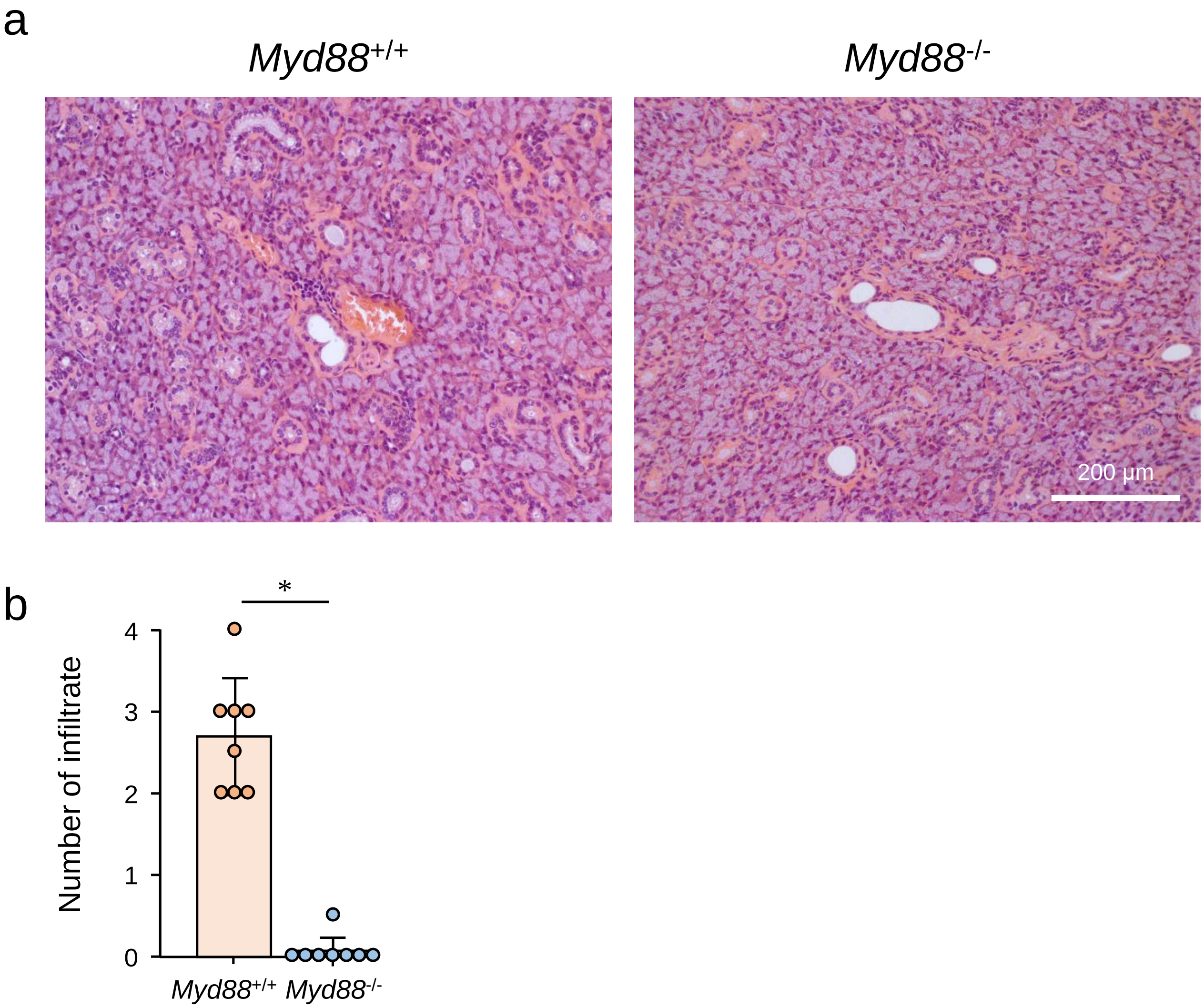

Supplementary Figure 6. Histology of SGs from wild-type and *Myd88*-deficient B6 mice.

(a) Representative areas of H&E-stained sections of SGs from 10-week-old female wild-type (left) and *Myd88*-deficient (right) B6 mice are shown. Original magnification: ×20. Additional images are shown in Fig. 3d.

(b) Number of lymphocytic infiltrates in SG sections evaluated from eight mice per genotype. Results are expressed as mean ± SD (bar graph) of mean values of total infiltrate numbers of four SG sections per animal (circle point). *, $p < 0.01$.

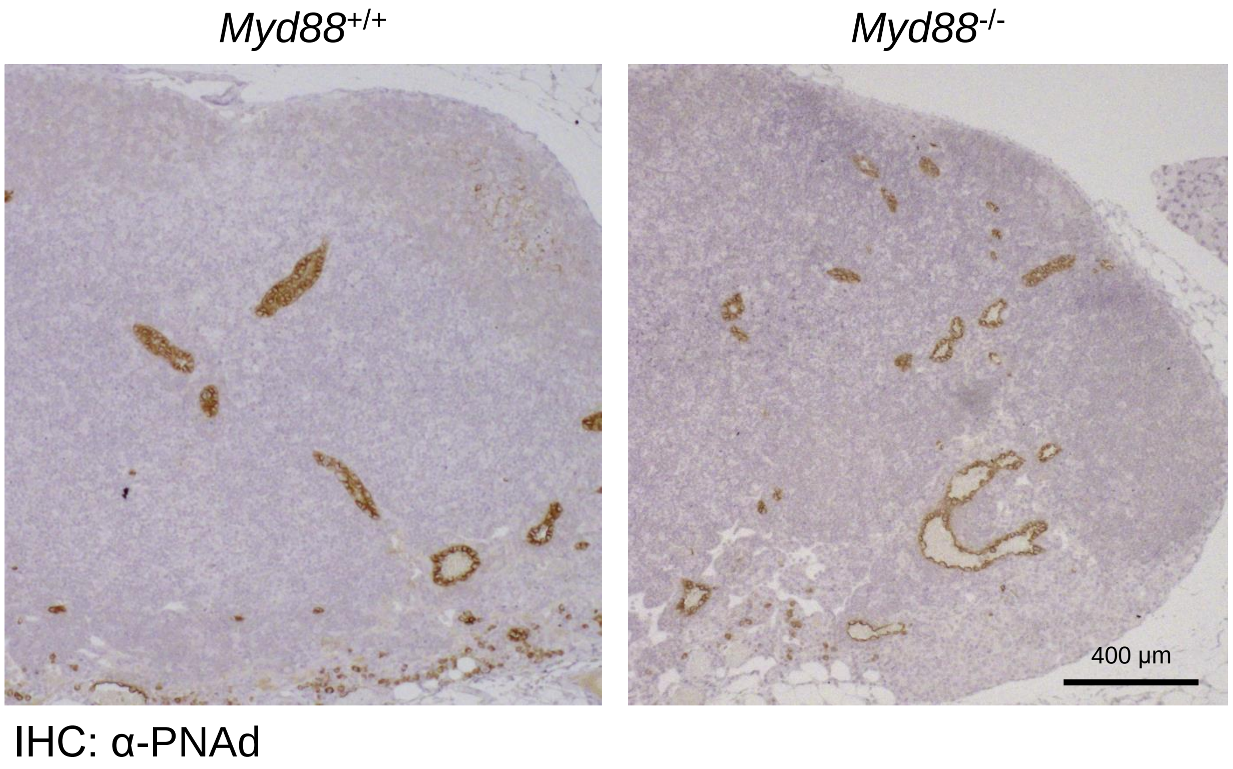

Supplementary Figure 7. Immunohistochemical staining of PNAd in SGALNs from wild-type and *Myd88*-deficient NOD mice.

Representative areas of anti-PNAd (MECA-79) antibody-stained sections of SGALNs from 12-week-old female wild-type (left) and *Myd88*-deficient (right) NOD mice are shown. Original magnification: ×10.

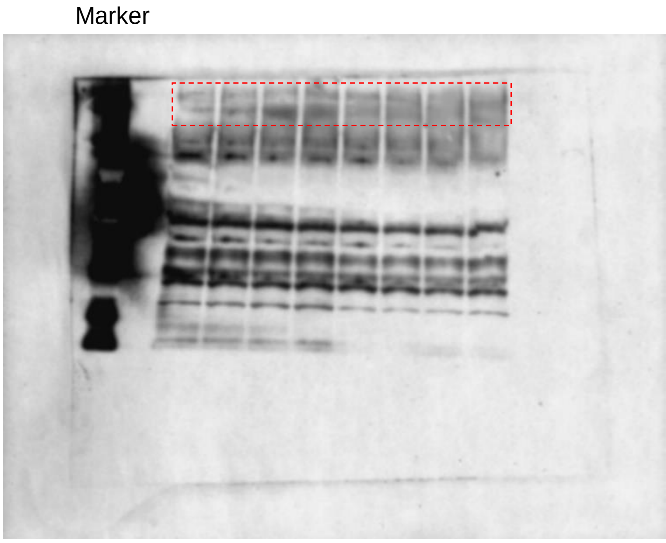

IB: α-NIK

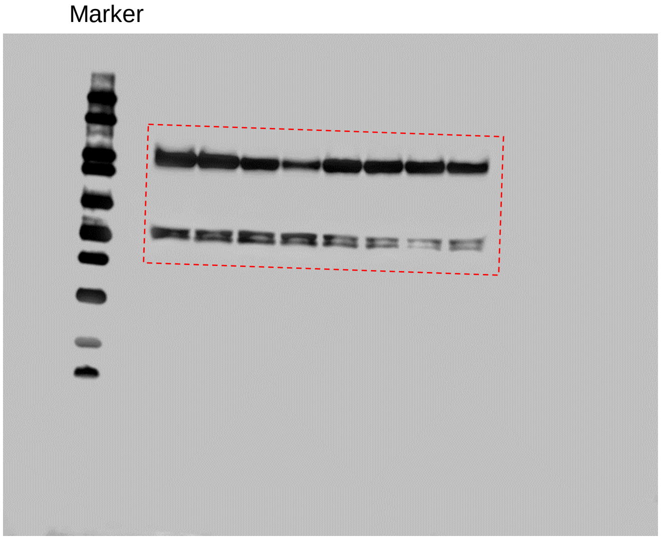

IB: α-NF-κB2 (p100/p52)

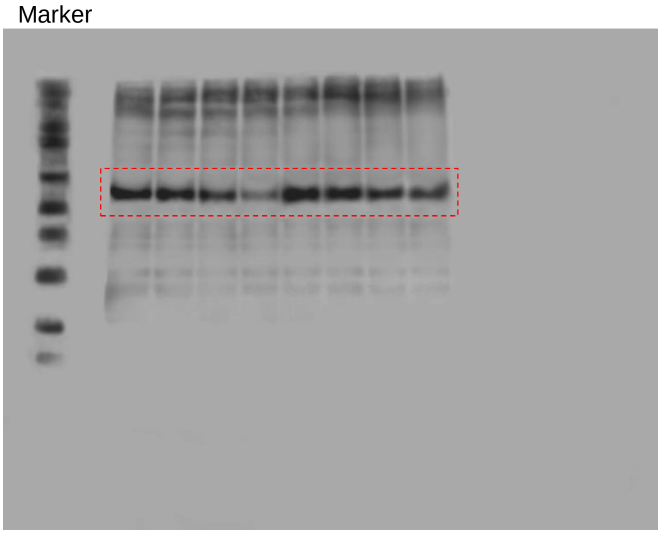

IB: α-TRAF3

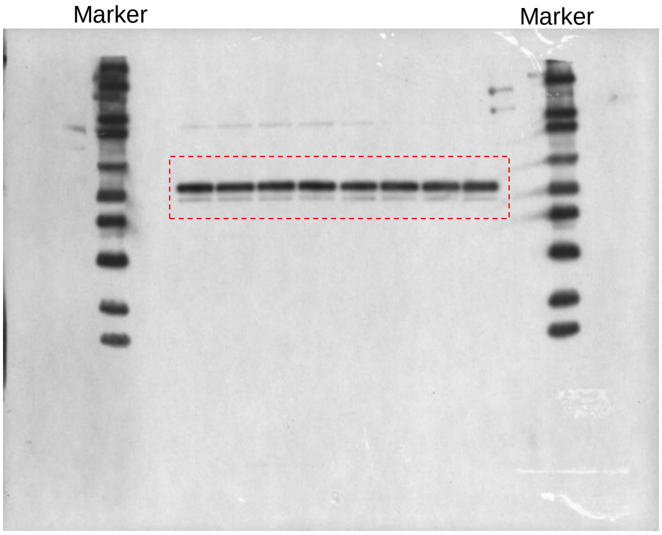

IB: α-α-Tubulin

Supplementary Figure 8. Uncropped images of immunoblot results of Fig. 6b.
The full-length blots, showing NIK, NF-κB2 (p100/p52), TRAF3, and α-tubulin, were obtained under the same experimental conditions. The cropped parts indicated by the boxes with red dashed lines are shown in Fig. 6b.

Supplementary Table 1. Complete list of the top 100 genes with the lowest P-value

Ranking	Systematic Name	Gene Name	Description	P-value	Fold change	Probe Name
1	NM_177823	Ubash3a	Mus musculus ubiquitin associated and SH3 domain containing, A (Ubash3a), mRNA [NM_177823]	4.078E-28	-2.58	A_51_P111532
2	NM_030098	Rnase6	Mus musculus ribonuclease, RNase A family, 6 (Rnase6), mRNA [NM_030098]	1.052E-26	-2.53	A_51_P104608
3	NM_134210	Vmn1r81	Mus musculus vomeronasal 1 receptor 81 (Vmn1r81), mRNA [NM_134210]	3.155E-26	2.55	A_51_P115229
4	NM_008134	Glycam1	Mus musculus glycosylation dependent cell adhesion molecule 1 (Glycam1), mRNA [NM_008134]	1.615E-23	-251.36	A_52_P602847
5	BC019489		Mus musculus immunoglobulin heavy chain 1a (serum IgG2a), mRNA (cDNA clone IMAGE:4011654). [BC019489]	1.885E-23	-109.13	A_55_P1956598
6	GQ984290		immunoglobulin heavy chain 3 (serum IgG2b) Gene [Source:MGI Symbol;Acc:MGI:96445] [ENSMUST00000103418]	2.342E-23	-31.01	A_55_P2063283
7			Unknown	2.374E-23	-48.47	A_51_P461067
8	XM_001472091	Igh-VJ558	predicted gene, 16844 Gene [Source:MGI Symbol;Acc:MGI:4439768] [ENSMUST00000103412]	2.522E-23	-138.03	A_55_P1960148
9	XM_003085465	Igh-VJ558	predicted gene, 16844 Gene [Source:MGI Symbol;Acc:MGI:4439768] [ENSMUST00000103412]	2.646E-23	-133.92	A_51_P509679
10	NM_152839	Igj	Mus musculus immunoglobulin joining chain (Igj), mRNA [NM_152839]	2.716E-23	-47.85	A_51_P150710
11	AM910933		immunoglobulin kappa chain, constant region Gene [Source:MGI Symbol;Acc:MGI:96495] [ENSMUST00000103410]	3.209E-23	-95.09	A_55_P2060897
12	NM_026976	Faim3	Mus musculus Fas apoptotic inhibitory molecule 3 (Faim3), mRNA [NM_026976]	3.617E-23	-89.24	A_51_P378298
13			Unknown	5.118E-23	-45.52	A_55_P2145224
14	NM_010389	H2-Ob	Mus musculus histocompatibility 2, O region beta locus (H2-Ob), mRNA [NM_010389]	5.177E-23	-80.88	A_55_P2082929
15	NM_009844	Cd19	Mus musculus CD19 antigen (Cd19), mRNA [NM_009844]	7.742E-23	-56.57	A_55_P2079079
16			Unknown	1.03E-22	-16.11	A_55_P2058040
17	NM_008339	Cd79b	Mus musculus CD79B antigen (Cd79b), mRNA [NM_008339]	1.048E-22	-35.12	A_51_P342652
18			Unknown	1.069E-22	-38.14	A_55_P2214124
19	AK080018		Mus musculus adult male aorta and vein cDNA, RIKEN full-length enriched library, clone:A530047M13 product:unclassifiable, full insert sequence. [AK080018]	1.11E-22	49.78	A_55_P2102881
20	NM_009835	Ccr6	Mus musculus chemokine (C-C motif) receptor 6 (Ccr6), transcript variant 1, mRNA [NM_009835]	1.132E-22	-14.86	A_55_P2108943
21			Unknown	1.221E-22	-31.10	A_51_P483576
22	AY170502		immunoglobulin lambda chain, constant region 2 Gene [Source:MGI Symbol;Acc:MGI:99547] [ENSMUST00000103749]	1.293E-22	-34.63	A_55_P2057283
23	NM_007641	Ms4a1	Mus musculus membrane-spanning 4-domains, subfamily A, member 1 (Ms4a1), mRNA [NM_007641]	1.439E-22	-71.84	A_52_P483336
24	NM_001164059	Sell	Mus musculus selectin, lymphocyte (Sell), transcript variant 2, mRNA [NM_001164059]	1.627E-22	-32.51	A_55_P2010152
25	NM_011177	Klk6	Mus musculus kallikrein related-peptidase 6 (Klk6), transcript variant 1, mRNA [NM_011177]	1.701E-22	-16.30	A_55_P2128591
26			lincRNA:chr5:107503800-107515575 reverse strand	1.905E-22	42.61	A_30_P01017987
27			lincRNA:chr4:89048647-89066563 forward strand	1.972E-22	-19.56	A_30_P01032359
28	XR_105809		immunoglobulin lambda chain, constant region 1 Gene [Source:MGI Symbol;Acc:MGI:99546] [ENSMUST00000103741]	2.554E-22	-18.91	A_55_P2111322
29			lincRNA:chr7:112934153-112943178 reverse strand	2.586E-22	-1.55	A_30_P01032903
30	NM_001037719	Btla	Mus musculus B and T lymphocyte associated (Btla), transcript variant 1, mRNA [NM_001037719]	2.738E-22	-30.60	A_55_P1964648
31			Unknown	2.786E-22	-16.71	A_55_P2290914
32	XM_003084474		Putative uncharacterized protein AI607873 [Source:UniProtKB/TrEMBL;Acc:D3Z2E7] [ENSMUST00000042610]	2.91E-22	-7.95	A_51_P325281
33	AK016784	4933412A08Rik	Mus musculus adult male testis cDNA, RIKEN full-length enriched library, clone:4933412A08 product:unclassifiable, full insert sequence. [AK016784]	3.231E-22	53.23	A_52_P204035
34	NM_027763	Treml1	Mus musculus triggering receptor expressed on myeloid cells-like 1 (Treml1), mRNA [NM_027763]	3.233E-22	-13.12	A_51_P279062
35			Unknown	3.531E-22	-22.62	A_55_P1977451
36			lincRNA:chr12:111617728-111665403 reverse strand	3.864E-22	-2.37	A_30_P01019746
37			Unknown	3.979E-22	-19.91	A_55_P2186978
38			Unknown	4.753E-22	-13.68	A_55_P2108311
39	NM_001162940	Gm10081	Mus musculus predicted gene 10081 (Gm10081), mRNA [NM_001162940]	4.872E-22	-6.82	A_55_P2104412
40			lincRNA:chr2:10237649-10287591 forward strand	4.924E-22	31.18	A_30_P01027645
41	NM_011681	Scgb1a1	Mus musculus secretoglobin, family 1A, member 1 (uteroglobin) (Scgb1a1), mRNA [NM_011681]	5.534E-22	-1.30	A_51_P128575
42			Unknown	5.677E-22	-9.06	A_55_P1987406
43	S77453		immunoglobulin heavy constant gamma 1 (G1m marker) Gene [Source:MGI Symbol;Acc:MGI:96446] [ENSMUST00000103421]	6.126E-22	-14.50	A_55_P2066173
44			lincRNA:chr12:33808493-33829143 forward strand	7.05E-22	45.40	A_30_P01030788
45	NM_009850	Cd3g	Mus musculus CD3 antigen, gamma polypeptide (Cd3g), mRNA [NM_009850]	7.28E-22	-7.02	A_55_P2072771
46	NM_013487	Cd3d	Mus musculus CD3 antigen, delta polypeptide (Cd3d), mRNA [NM_013487]	7.51E-22	-22.39	A_51_P285206
47			Unknown	7.995E-22	-13.08	A_55_P2058023
48	NM_007719	Ccr7	Mus musculus chemokine (C-C motif) receptor 7 (Ccr7), mRNA [NM_007719]	8.299E-22	-21.11	A_55_P2023542
49	NM_009394	Tnnc2	Mus musculus troponin C2, fast (Tnnc2), mRNA [NM_009394]	1.289E-21	-115.03	A_51_P353232
50			lincRNA:chr13:59245617-59245945 reverse strand	1.32E-21	-12.28	A_30_P01031668
51	NM_010689	Lat	Mus musculus linker for activation of T cells (Lat), mRNA [NM_010689]	1.374E-21	-4.19	A_51_P495242
52	NM_007551	Cxcr5	Mus musculus chemokine (C-X-C motif) receptor 5 (Cxcr5), mRNA [NM_007551]	1.659E-21	-12.52	A_52_P70796
53			lincRNA:chr11:63054652-63131714 reverse strand	1.819E-21	-4.21	A_30_P01030688
54	NM_001110276	Itsn1	Mus musculus intersectin 1 (SH3 domain protein 1A) (Itsn1), transcript variant 3, mRNA [NM_001110276]	2.028E-21	30.69	A_55_P1958062
55	NM_001011810	Olfr485	Mus musculus olfactory receptor 485 (Olfr485), mRNA [NM_001011810]	2.08E-21	21.86	A_51_P147188
56	AF045501		predicted gene, 16746 Gene [Source:MGI Symbol;Acc:MGI:4439670] [ENSMUST00000109705]	2.092E-21	-9.33	A_52_P213483
57	U07661		T-cell receptor beta-2 chain C region [Source:UniProtKB/Swiss-Prot;Acc:P01851] [ENSMUST00000103299]	2.834E-21	-13.21	A_51_P478172
58	U58290	LOC639930	Mus musculus J558+ IgM heavy chain mRNA, partial cds. [U58290]	2.86E-21	-18.39	A_55_P2065506
59			lincRNA:chr15:38639259-38639546 reverse strand	3.949E-21	-1.31	A_30_P01023385
60	NM_007650	Cd5	Mus musculus CD5 antigen (Cd5), mRNA [NM_007650]	4.46E-21	-9.60	A_51_P167263
61	NM_133809	Kmo	Mus musculus kynurenine 3-monooxygenase (kynurenine 3-hydroxylase) (Kmo), mRNA [NM_133809]	4.461E-21	-5.40	A_55_P2145606
62	M57280		predicted gene, 16939 Gene [Source:MGI Symbol;Acc:MGI:4439863] [ENSMUST00000103320]	4.911E-21	-12.16	A_51_P390937
63	NM_145141	Fcrla	Mus musculus Fc receptor-like A (Fcrla), transcript variant 2, mRNA [NM_145141]	6.195E-21	-24.52	A_51_P154485
64	NM_019866	Spib	Mus musculus Spi-B transcription factor (Spi-1/PU.1 related) (Spib), mRNA [NM_019866]	7.537E-21	-23.98	A_55_P2113703
65	X75105		predicted gene, 16637 Gene [Source:MGI Symbol;Acc:MGI:4439561] [ENSMUST00000103328]	7.733E-21	-6.94	A_55_P2047589
66	NM_001033791	Gm12888	Mus musculus predicted gene 12888 (Gm12888), mRNA [NM_001033791]	7.937E-21	10.88	A_66_P133702
67	X59099		predicted gene 1502 Gene [Source:MGI Symbol;Acc:MGI:2686348] [ENSMUST00000103348]	8.623E-21	-22.83	A_55_P2103948
68	XM_890897		Androgen-binding protein Fragment [Source:UniProtKB/TrEMBL;Acc:D2XZ31] [ENSMUST00000108097]	8.646E-21	12.02	A_55_P2022288
69			lincRNA:chr5:113594167-113645342 reverse strand	9.822E-21	-1.77	A_30_P01021739
70			Unknown	9.911E-21	-1.64	A_55_P2144961
71			lincRNA:chr15:51829797-51844247 reverse strand	1.271E-20	-1.54	A_30_P01020668
72	NM_031247	Gimap3	Mus musculus GTPase, IMAP family member 3 (Gimap3), nuclear gene encoding mitochondrial protein, mRNA [NM_031247]	1.301E-20	-13.21	A_55_P2003221
73	BC117076	1700014N06Rik	Mus musculus RIKEN cDNA 1700014N06 gene, mRNA (cDNA clone MGC:151453 IMAGE:40126395), complete cds. [BC117076]	1.305E-20	-16.16	A_55_P1958047
74			Unknown	1.372E-20	31.57	A_55_P2131742
75	NM_001163810	1700008P20Rik	Mus musculus RIKEN cDNA 1700008P20 gene (1700008P20Rik), mRNA [NM_001163810]	1.465E-20	1.79	A_51_P128075
76			Unknown	1.59E-20	-7.50	A_52_P320761
77	AK007918		immunoglobulin heavy chain 1b (serum IgG2c) Gene [Source:MGI Symbol;Acc:MGI:2686979] [ENSMUST00000103416]	1.656E-20	-13.09	A_55_P2034655
78	NM_001009952	Abpd	Mus musculus androgen binding protein delta (Abpd), mRNA [NM_001009952]	1.717E-20	9.25	A_55_P2057004
79	NM_001033344	Dusp27	Mus musculus dual specificity phosphatase 27 (putative) (Dusp27), transcript variant 1, mRNA [NM_001033344]	1.897E-20	-10.34	A_51_P235088
80	NM_013503	Drd5	Mus musculus dopamine receptor D5 (Drd5), mRNA [NM_013503]	2.281E-20	-13.58	A_52_P35384
81	NM_010090	Dusp2	Mus musculus dual specificity phosphatase 2 (Dusp2), mRNA [NM_010090]	2.625E-20	-10.08	A_52_P399934
82	NM_028075	Tnfrsf13c	Mus musculus tumor necrosis factor receptor superfamily, member 13c (Tnfrsf13c), mRNA [NM_028075]	2.741E-20	-21.60	A_55_P2117614
83	NM_001037800	Cd209b	Mus musculus CD209b antigen (Cd209b), transcript variant 2, mRNA [NM_001037800]	2.822E-20	-9.79	A_52_P267717
84	NM_009405	Tnni2	Mus musculus troponin I, skeletal, fast 2 (Tnni2), mRNA [NM_009405]	3.009E-20	-55.61	A_55_P2040893
85	NM_023483	1110032A03Rik	Mus musculus RIKEN cDNA 1110032A03 gene (1110032A03Rik), mRNA [NM_023483]	3.377E-20	-1.67	A_55_P2023290
86			Unknown	3.403E-20	-9.73	A_55_P1999715
87			lincRNA:chr14:52864224-52878449 forward strand	3.492E-20	-2.51	A_30_P01023608
88	NM_001081369	Ccdc153	Mus musculus coiled-coil domain containing 153 (Ccdc153), mRNA [NM_001081369]	3.924E-20	-3.49	A_52_P352735
89	NM_198635	Gm5134	Mus musculus predicted gene 5134 (Gm5134), mRNA [NM_198635]	4.334E-20	-1.20	A_52_P235880
90			lincRNA:chr14:33895294-33923644 reverse strand	4.533E-20	-1.03	A_30_P01031245
91	NM_001037906	Nell1	Mus musculus NEL-like 1 (chicken) (Nell1), mRNA [NM_001037906]	4.728E-20	4.90	A_55_P1967500
92			lincRNA:chr11:18479852-18485704 forward strand	4.96E-20	-7.45	A_30_P01027061
93			lincRNA:chr9:27148989-27155714 forward strand	5.186E-20	16.23	A_30_P01023937
94	NM_013645	Pvalb	Mus musculus parvalbumin (Pvalb), mRNA [NM_013645]	6.073E-20	-161.02	A_55_P2059040
95	AY174043		Mus musculus clone BaPB1-61 immunoglobulin heavy chain variable region mRNA, partial cds. [AY174043]	6.089E-20	-9.04	A_55_P2001262
96	AF296427	LOC640979	Mus musculus clone J558.B10 immunoglobulin heavy chain variable region mRNA, partial cds. [AF296427]	6.532E-20	-25.64	A_51_P513776
97	NR_028030	Gm9767	Mus musculus predicted gene 9767 (Gm9767), non-coding RNA [NR_028030]	7.202E-20	-2.16	A_51_P247496
98			lincRNA:chr15:79764481-79809909 reverse strand	7.933E-20	-1.29	A_30_P01030130
99			lincRNA:chr15:38630866-38640611 reverse strand	8.909E-20	-0.06	A_30_P01026218
100	NM_011414	Slpi	Mus musculus secretory leukocyte peptidase inhibitor (Slpi), mRNA [NM_011414]	8.944E-20	-10.58	A_52_P472324
